# Zinc-Induced Transposition of Insertion Sequence Elements Contributes to Increased Adaptability of *Cupriavidus metallidurans*

**DOI:** 10.3389/fmicb.2016.00359

**Published:** 2016-03-23

**Authors:** Joachim Vandecraen, Pieter Monsieurs, Max Mergeay, Natalie Leys, Abram Aertsen, Rob Van Houdt

**Affiliations:** ^1^Unit of Microbiology, Belgian Nuclear Research Centre (SCK•CEN)Mol, Belgium; ^2^Laboratory of Food Microbiology and Leuven Food Science and Nutrition Research Centre, Department of Microbial and Molecular Systems, Faculty of Bioscience Engineering, Katholieke Universiteit LeuvenLeuven, Belgium

**Keywords:** metal ions, resistance, *Cupriavidus*, insertion sequence element, adaptation

## Abstract

Bacteria can respond to adverse environments by increasing their genomic variability and subsequently facilitating adaptive evolution. To demonstrate this, the contribution of Insertion Sequence (IS) elements to the genetic adaptation of *Cupriavidus metallidurans* AE126 to toxic zinc concentrations was determined. This derivative of type strain CH34, devoid of its main zinc resistance determinant, is still able to increase its zinc resistance level. Specifically, upon plating on medium supplemented with a toxic zinc concentration, resistant variants arose in which a compromised *cnrYX* regulatory locus caused derepression of CnrH sigma factor activity and concomitant induction of the corresponding RND-driven *cnrCBA* efflux system. Late-occurring zinc resistant variants likely arose in response to the selective conditions, as they were enriched in *cnrYX* disruptions caused by specific IS elements whose transposase expression was found to be zinc-responsive. Interestingly, deletion of *cnrH*, and consequently the CnrH-dependent adaptation potential, still enabled adaptation by transposition of IS elements (IS*Rme5* and IS*1086*) that provided outward-directed promoters driving *cnrCBAT* transcription. Finally, adaptation to zinc by IS reshuffling can also enhance the adaptation to subsequent environmental challenges. Thus, transposition of IS elements can be induced by stress conditions and play a multifaceted, pivotal role in the adaptation to these and subsequent stress conditions.

## Introduction

Bacteria continuously evolve to survive different environmental challenges and the field of evolutionary biology has long been interested in the interplay between generation of genetic diversity and natural selection favoring the best adapted organism. The central dogma that evolution proceeds through natural selection of heritable mutations was first proposed by Darwin (Darwin, 1910). In addition, however, Darwin also suggested that environmental stress might generate a diversity of genetic variants, upon which subsequent natural selection works (Darwin, 1910). The claim that mutation rate might be influenced by stress is, however, still under debate (Rosenberg and Hastings, 2004; Roth, 2011). The classic experiments of Luria and Delbrück (1943) and Lederberg (1989) demonstrated for the first time that mutations can arise without the influence of selective stress. However, the lethal selections they used could likely not have detected mutations induced by selective conditions, and others have described genetic systems in which selective conditions indeed seemed to increase the mutation rate (Shapiro, 1984; Cairns et al., 1988; Hall, 1990; Foster, 2007; MacLean et al., 2013; Ram and Hadany, 2014). In addition, the coupling of potential mutation-generating systems to local events such as stress-induced transcription of a specific subset of genes (Wright, 2004) might form an example of stress-directed mutation where the mutation is specifically induced by the stress condition that the mutation relieves (Cairns et al., 1988). However, non-selected mutations readily occur during selective conditions disputing the existence of non-random mutations (Foster, 2007).

One frequently encountered type of mutation results from the hopping of transposable elements from the donor to the target site, potentially leading to important phenotypic changes (Mahillon and Chandler, 1998; Mahillon et al., 1999). Insertion sequence (IS) elements, which are widely distributed in the genomes of bacteria as well as higher organisms (Siguier et al., 2006, 2014), are of interest as they constitute an important driving force for genome plasticity (Kazazian, 2004). On the one hand, intra-genomic transposition of these small elements (<2.5 kb), which generally encode no other functions than those involved in their mobility, can both inactivate or activate adjacent genes by physically interrupting coding sequences or encoding regulatory sequences, respectively. The activation can be caused by a promoter encoded by the element and directed outward to the adjacent gene (Ciampi et al., 1982; Zhang and Saier, 2009; Wang and Wood, 2011; Saier and Zhang, 2014) or by the formation of a hybrid promoter in which the −35 region at the end of the IS element is correctly situated close to the −10 region of the adjacent gene (Jaurin and Normark, 1983; Prentki et al., 1986). On the other hand, homologous IS elements within the chromosome can become substrates of the cell's recombination machinery, which can result in complex chromosomal rearrangements (e.g., inversions, duplications, or deletions) depending on the orientation of the involved IS elements (Louarn et al., 1985; Schneider et al., 2000a; Parkhill et al., 2003).

Selective conditions can promote the activation and redistribution of IS elements that exist in the chromosome. Indeed, stress factors were found to induce the transposition of mobile genetic elements (MGEs) in plants (McClintock, 1984; Studer et al., 2011), flies (González et al., 2010), yeast (Stanley et al., 2010), and bacteria (Ohtsubo et al., 2005; Drevinek et al., 2010). The acquisition of phenotypic traits caused by transposition of IS elements has been well-documented, particularly in bacteria. Different conditions such as UV light, low frequency magnetic fields or sub-inhibitory concentrations of antibiotics were shown to promote transposition of IS elements in *Escherichia coli* (Eichenbaum and Livneh, 1998; Del Re et al., 2003; Lartigue et al., 2006). Transposition of IS elements in response to a selective condition has also been described in other gram-negative genera, e.g., *Pseudomonas* and *Burkholderia* in response to high temperature, conjugation or oxidative stress (Ohtsubo et al., 2005; Christie-Oleza et al., 2009; Drevinek et al., 2010). Active redistribution of IS elements was also observed in the gram-positive *Deinococcus radiodurans* after exposure to radiation (Pasternak et al., 2010) and in the gram-positive *Bacillus subtilis* under competence-inducing conditions (Takahashi et al., 2007).

*Cupriavidus metallidurans* strains are, next to metal-contaminated soils (Diels and Mergeay, 1990; Brim et al., 1999; Goris et al., 2001), being increasingly recovered from other anthropogenic environments not typified by metal contamination (Van Houdt et al., 2012; Mijnendonckx et al., 2013), including medically-relevant sources (Coenye et al., 2005; Langevin et al., 2011; D'Inzeo et al., 2015). They are studied for their resistance and adaptation to toxic levels of metal ions. Type strain *C*. *metallidurans* CH34 counteracts metal ion toxicity via a battery of resistance mechanisms, including transporters belonging to the Resistance Nodulation cell Division (RND), the cation diffusion facilitator (CDF) and the P-type ATPase families, with an important role for its two mega-plasmids as most of the heavy metal resistance mechanisms are encoded on either pMOL28 or pMOL30 (Mergeay et al., 1985, 2003; Nies and Silver, 1995; Nies, 1999, 2003; Monchy et al., 2007; von Rozycki and Nies, 2009; Janssen et al., 2010). In addition, *C. metallidurans* CH34 harbors 21 distinct IS elements belonging to 10 different IS families (Mijnendonckx et al., 2011). Out of the 21 distinct IS elements, active transposition has been observed for IS*Rme1*, IS*Rme3*, IS*1086*, IS*1087B*, IS*1088*, and IS*1090* (Dong et al., 1992; Collard et al., 1993; Grass et al., 2000; Ma-e-Talat, 2000; Schneider et al., 2000b; Tibazarwa et al., 2000). In fact, active involvement of IS*1087B* in metal resistance has been shown for *C*. *metallidurans* AE126, which is a derivative of CH34 sensitive to zinc as it only carries plasmid pMOL28 and not plasmid pMOL30 (Collard et al., 1993; Tibazarwa et al., 2000). Spontaneous zinc-resistant AE126 mutants constitutively expressing *cnrCBAT* coding for the RND-driven efflux system CnrCBAT appear when AE126 is grown in the presence of 0.8 mM Zn^2+^ (Collard et al., 1993; Grass et al., 2000; Tibazarwa et al., 2000). The *cnrCBAT* operon is regulated by the upstream *cnrYXH* locus coding for the membrane-bound anti-sigma factor CnrY, the sensor protein CnrX, and the ECF (Extracytoplasmic Function) family sigma factor CnrH. The latter is released from the CnrYX transmembrane anti-sigma factor complex in the presence of inducers (Ni^2+^ or Co^2+^) and subsequently promotes transcription of both *cnrYXH* and *cnrCBAT* (Grosse et al., 2007; Trepreau et al., 2011, 2014; Maillard et al., 2015). Analysis of two of such derivatives indicated that the inactivation of the gene coding for the anti-sigma factor CnrY (by IS*1087B* and a frameshift mutation), and concomitant release of CnrH increased transcription of the structural *cnrCBAT* cluster and led to increased (non-specific) Zn^2+^ efflux (Collard et al., 1993; Grass et al., 2000; Tibazarwa et al., 2000).

These observations indicate that IS transposition may play a dynamic role in the genetic adaptation of *C. metallidurans* AE126 to toxic zinc concentrations. Therefore, in this study, we thoroughly scrutinized the mutations leading to, and the genetic determinants essential for increased zinc resistance in AE126. The involvement of IS elements was dissected via the identification of the concerned IS elements, their target sites, and the effect elicited by their transposition as well as via analysis of transposase expression upon metal ion exposure.

## Materials and methods

### Strains, media, and culture conditions

Bacterial strains and plasmids used in this study are listed in Table 1. *C*. *metallidurans* strains were routinely cultured at 30°C in Tris-buffered mineral medium supplemented with 0.2% (w/v) sodium gluconate (MM284) as described previously (Mergeay et al., 1985). *E*. *coli* strains were routinely cultured at 37°C in Lysogeny Broth (LB). Liquid cultures were grown in the dark on a rotary shaker at 150 rpm, for culturing on agar plates 2% agar (Thermo Scientific, Oxoid) was added. When appropriate, the following chemicals (Sigma-Aldrich or Thermo Scientific) were added to the growth medium at the indicated final concentrations: kanamycin [50 μg/ml for *E. coli* (Km^50^) or 1500 μg/ml for *C*. *metallidurans* (Km^1500^)], tetracycline (20 μg/ml), chloramphenicol (30 μg/ml), Zn^2+^ (0.1 or 0.8 mM; as zinc sulfate heptahydrate), Ni^2+^ (0.1 mM; as nickel chloride hexahydrate), Cd^2+^ (0.01 mM; as cadmium chloride hemipentahydrate), Co^2+^ (0.05 mM; as cobalt chloride hexahydrate), 5-bromo-4-chloro-3-indolyl-β-galactopyranoside (X-Gal; 40 μg/ml), and isopropyl β-D-1-thiogalactopyranoside (IPTG; 0.1 mM).

**Table 1:** **Strains and plasmids used in this study**.

**Strain or plasmid**	**Genotype/Relevant characteristics**	**Reference**
**STRAIN**		
***C***. ***Metallidurans***		
AE126	pMOL28 *cnrYXHCBAT*, Zn^S^	(Mergeay et al., 1985)
AE126^R1^	Nonsense mutation in *cnrX*, 2d	This study
AE126^R2^	Frameshift, 1-bp deletion in *cnrX*, 5d	This study
AE126^R3^	Frameshift, 5-bp duplication in *cnrY*, 5d	This study
AE126^R4^	IS*Rme5* insertion in *cnrX*, 5d	This study
AE126 *ΔcnrH::tet*	Deletion of *cnrH*, Tc^R^	This study
AE126 *ΔcnrH::tet*^R1^	IS*Rme5* insertion between *ΔcnrH::tet* and *cnrC*	This study
AE126 *cnrH::tet*^R2^	IS*1086* insertion between *ΔcnrH::tet* and *cnrC*	This study
AE104	Ni^S^ Co^S^ Zn^S^	(Mergeay et al., 1985)
***E. coli***		
DG1	*mcrA* Δ(*mrr-hsdRMS-mcrBC*, modification-, restriction-) ϕ80*lacZ*ΔM15 Δ*lacX74 recA1 araD139* Δ*(ara-leu)7697 galU galK rpsL endA1 nupG*	Eurogentec (Belgium)
HB101	F^−^*mcrB mrr hsdS20(rB- mB-) recA13 leuB6 ara-14 proA2 lacY1 galK2 xyl-5 mtl-1 rpsL20 glnV44* λ-	Laboratory collection
**PLASMID**		
pRK600	Helper plasmid; Cm^R^ *tra*	Laboratory collection
pK18mob	*lacZα* Km^R^ *oriT oriV*	(Katzen et al., 1999)
pK18mob-*cnrH*	*cnrH* Km^R^	This study
pK18mob-*cnrH::tet*	*cnrH*::tet Tc^R^ Km^R^	This study
pJV240	IncQ *sacBR*, Km^R^	(Dong et al., 1992)
pBBR1MCS2	*ori* pBBR1 *oriT lacZα*, Km^R^	(Kovach et al., 1995)
pBBR1MCS2-*cnrYXH*	*cnrYXH* of AE126, Ni^S^ Co^S^ Zn^S^	This study
pBBR1MCS2-*cnrYXH*^R^	*cnrYXH* of AE126^R1^, Ni^S^ Co^S^ Zn^S^	This study
pBBR1MCS2-*cnrYXHCBAT*	*cnrYXHCBAT* of AE126, Ni^R^ Co^R^ Zn^S^	This study
pBBR1MCS2-*cnrYXHCBAT*^R^	*cnrYXHCBAT* of AE126^R1^, Ni^R^ Co^R^ Zn^R^	This study
pBBR1MCS2-*cnrYXΔH::tetCBAT*	Deletion of *cnrH*, Ni^S^ Co^S^ Zn^S^ Tc^R^	This study
pBBR1MCS2-*cnrYXΔH::tetCBAT*^R^	*cnrYXΔH::tetCBAT*^R^ of AE126 *ΔcnrH::tet*^R1^, Ni^S^ Co^S^ Zn^R^ Tc^R^	This study
pGLR1	Km^R^ *ori* pBBR1 *oriT*; GFP-*luxCDABE* reporter	(Benedetti et al., 2012)
pGLR1-P_IS_*_*Rme*5_*-*gfpluxCDABE*	Promoter IS*Rme5* (91 bp)	This study
pGLR1-P_IS_*_*Rme*5_*_a_-*gfpluxCDABE*	Promoter IS*Rme5*a (Rmet_1251; 209 bp)	This study
pGLR1-P_IS_*_*Rme*5_*_b_-*gfpluxCDABE*	Promoter IS*Rme5*b (Rmet_1280; 257 bp)	This study
pGLR1-P_IS_*_*Rme*5_*_c_-*gfpluxCDABE*	Promoter IS*Rme5*c (Rmet_1301; 297 bp)	This study
pGLR1-P_IS_*_*Rme*5_*_d_-*gfpluxCDABE*	Promoter IS*Rme5*d (Rmet_4152; 239 bp)	This study
pGLR1-P_IS_*_1088_*-*gfpluxCDABE*	Promoter IS*1088* (85 bp)	This study
pGLR1-P_IS_*_1087*B*_*-*gfpluxCDABE*	Promoter IS*1087B* (89 bp)	This study
pGLR1-P*_*cnrC*_*-*gfpluxCDABE*	Native *cnrC* promoter AE126	This study
pGLR1-P_IS_*_*Rme*5*cnrC*_*-*gfpluxCDABE*	Fragment *cnrC* promoter AE126 *ΔcnrH::tet*^R1^ (including 3′ IS*Rme5* fragment without its endogenous transposase promoter)	This study
pGLR1-P_IS_*_1086*cnrC*_*-*gfpluxCDABE*	Fragment *cnrC* promoter AE126 *ΔcnrH::tet*^R2^ (including 3′ IS*1086* fragment without its endogenous transposase promoter)	This study

*^S^sensitive, ^R^resistant, 2d, and 5d: isolated from the first and second population after 2 and 5 days of exposure, respectively*.

### Determination of growth and minimal inhibitory concentration

The susceptibility of *C*. *metallidurans* strains to different heavy metals was determined via minimal inhibitory concentrations (MICs). The strains were cultivated in triplicate by inoculating 2 ml MM284 supplemented with different concentrations of heavy metals with 20 μL of a stationary phase culture at 30°C. The MIC is defined as the lowest concentration of heavy metal that will inhibit visible growth of the culture after 2 days of incubation. Growth experiments were performed by 1:100 dilution of stationary phase cultures in fresh MM284 and subsequently for each sample (biological replicates), 200 μL was added to a 96-well white cell culture plate (Nunc Flat-bottom, Thermo Scientific) which was placed into a CLARIOstar® (BMG LABTECH). Plates were incubated at 30°C with shaking for 48 h and at 30-min time intervals, the optical density at 600 nm was measured. The growth data were fitted by the model of Baranyi and Roberts (1994) using the Microsoft Excel add-in package DMFit (Institute of Food Research, Norwich, United Kingdom). Statistical comparison of the growth curve parameters was performed using a one-way ANOVA analysis, followed by a *post-hoc* Tukey test.

### Isolation of zinc-resistant mutants

*C*. *metallidurans* AE126 was cultivated in MM284 at 30°C up to stationary phase, 10^9^ cells were pelleted and cell suspensions (100 μL) of a serial ten-fold dilution in saline were spread on MM284 agar plates containing a final concentration of 0.8 mM Zn^2+^ and incubated at 30°C. Colony forming units (CFU) were counted after day 2 up to day 5 and survival frequency was calculated as viable cell count on MM284 0.8 mM Zn^2+^ agar plates divided by viable cell count on MM284 agar plates. The variance-to-mean ratio and mutation rate in a fluctuation assay with 20 independent cultures was calculated according to Luria and Delbrück (1943) and using Fluctuation Analysis CalculatOR (FALCOR) (Hall et al., 2009), respectively.

### Analysis of IS transposition

Transposition of IS elements in the regulatory locus of the *cnr* operon (Supplementary Figure 1) was identified by colony PCR (DreamTaq DNA polymerase, Thermo Scientific) with primers CnrFw and CnrRv (Supplementary Table 1) and sequencing. A *sacBR* IS trap (Gay et al., 1985; Dong et al., 1992) was used to compare the adaptation potential of AE126^R4^, which carries an IS*Rme5* copy inserted in *cnrX* in the same transcriptional orientation as *cnrYX*, with AE126. Sucrose-resistant derivatives of *C. metallidurans* AE126 and AE126^R4^ carrying pJV240 (Gay et al., 1985; Dong et al., 1992) were selected on LB plates without NaCl and supplemented with 10% (w/v) sucrose and 1500 μg/ml kanamycin. The insertion of IS*Rme5* in pJV240 was identified by colony PCR with primer pairs IS*Rme5*_Fw and SacR_Rv or pIS*Rme5*_Rv and SacR_Rv, respectively (Supplementary Table 1).

### Whole genome gene expression microarrays

The whole-genome expression profile of AE126^R2^ and AE126^R3^ were compared to its parental strain AE126 by whole genome oligonucleotide microarrays. The strains were cultivated by inoculating 30 ml of MM284 in biological triplicates with 300 μL of a stationary phase culture at 30°C. These subcultures were allowed to grow until an OD_600_-value of around 0.6 was reached. Next, cells were harvested by centrifugation for 2 min at 10,000 rpm and the bacterial pellets were flash frozen by immersion into liquid nitrogen. The bacterial pellets were kept frozen at −80 °C until total RNA extraction was performed using the SV Total RNA Isolation system (Promega Corporation). The quantity of extracted RNA was measured using a NanoDrop™ 1000 spectrophotometer (Thermo Scientific). The RNA quality was determined with a Bioanalyzer (Agilent 2100 Electrophoresis Bioanalyzer Agilent Technologies). Only extracted RNA with a RNA integrity number (RIN number) of higher than eight was accepted for further analysis (Schroeder et al., 2006). Twenty micrograms of RNA were reverse transcribed following the instructions provided with the Pronto kit (Promega). Microarray slide spotting, RNA labeling, hybridization, and data analysis were performed as previously described (Monsieurs et al., 2011). The full description of the microarray data have been deposited at the Gene Expression Omnibus website (http://www.ncbi.nlm.nih.gov/geo/) under accession number GSE74091.

### *cnrH* mutant deletion construction

The *cnrH* gene of *C*. *metallidurans* AE126 was amplified by PCR (Phusion High-Fidelity DNA polymerase) with primer pairs cnrH_Fw-Rv (Supplementary Table 1), providing *Hind*III/*Eco*RI restriction sites. Afterwards, these PCR products were cloned as a *Hind*III/*Eco*RI fragment into the mobilizable suicide vector pK18mob. The resulting pK18mob *cnrH* plasmid from an *E. coli* DG1 transformant selected on LB Km^50^ was further confirmed by sequencing prior to amplifying of the flanking sequences of *cnrH* by inverse PCR (Phusion High-Fidelity DNA polymerase) with primer pair cnrH_tet_Fw-Rv (Supplementary Table 1), providing *Bcu*I/*Xba*I restriction sites. At the same time the *tet* gene from pACYC184 (Table 1; Chang and Cohen, 1978) was amplified by PCR (Phusion High-Fidelity DNA polymerase) with primer pair Tet_Fw-Rv (Supplementary Table 1), providing *Bcu*I/*Xba*I restriction sites. Afterwards, this PCR product was cloned as a *Bcu*I/*Xba*I fragment into the former inverse *cnrH* PCR product. The resulting pK18mob-*cnrH::tet* plasmid from an *E. coli* DG1 transformant selected on LB Tc^20^ Km^50^ was further confirmed by sequencing prior to conjugation (triparental with *E. coli* HB101 pRK600 as helper) to *C. metallidurans* AE126. The resulting transformants selected on MM284 Km^1500^ were replica plated on MM284 Tc^20^ and MM284 Km^1500^. AE126Δ*cnrH::tet* cells resistant to Tc^20^ but sensitive to Km^1500^ were further confirmed by sequencing.

### Identification of *cnrCBAT* transcription start site

The transcription start site of *cnrCBAT* in wild type AE126 and in the two zinc-resistant derivatives AE126Δ*cnrH::tet*^R1^ and AE126Δ*cnrH::tet*^R2^ were identified using the cRACE method (Maruyama et al., 1995; Dallmeier and Neyts, 2013). The strains were cultivated by inoculating 30 ml of MM284 with 300 μL of a stationary phase *C. metallidurans* AE126, AE126Δ*cnrH::tet*^R1^, or AE126Δ*cnrH::tet*^R2^ culture at 30°C. The cultures were allowed to grow until an OD_600_-value of around 0.6 was reached. Next, cells were harvested by centrifugation for 2 min at 10,000 rpm and the bacterial pellets were flash frozen by immersion into liquid nitrogen. The bacterial pellets were kept frozen at −80°C until total RNA extraction was performed using the SV Total RNA Isolation system (Promega Corporation). The quantity of extracted RNA was measured using a NanoDrop™ 1000 spectrophotometer (Thermo Scientific). The RNA quality was determined with a Bioanalyzer (Agilent 2100 Electrophoresis Bioanalyzer Agilent Technologies). Only extracted RNA with a RNA integrity number (RIN number) of higher than eight was accepted for further analysis (Schroeder et al., 2006). One microgram of RNA was reverse transcribed following the instructions provided with the GoScript™ Reverse Transcription System (Promega) with a gene-specific 5′-phosphorylated oligonucleotide cnrC_R1 (Supplementary Table 1). Subsequently, three volumes of TE buffer (10 mM Tris-HCl and 1 mM ethylenediaminetetraacetic acid, pH 8.0) containing 4 μg/ml RNase A (Promega) was added to the reaction. Successively, 28 μL of the resulting cDNAs was circularized by 20 U of T4 RNA ligase (Thermo Scientific) in the presence of 15% (w/v) polyethylene glycol (PEG4000; Thermo Scientific) in a total volume of 50 μL at 37°C for 60 min. Next 1.5 U of Pfu DNA polymerase (Thermo Scientific) was added to the reaction at 37°C for 30 min employing its 3′-5′ exonuclease activity to remove unreacted residual first-strand primers and cDNAs. Finally, a 2 μL aliquot was directly used as template for the amplification by PCR (DreamTaq DNA polymerase) with primer pair cnrC_F1-R2 (Supplementary Table 1), the PCR product was cloned by TOPO cloning (Thermo Scientific) and the transcription start site was identified by sequencing.

### Construction of plasmids

The *cnr* locus (*cnrYXHCBAT*) and its regulatory part (*cnrYXH*) from *C*. *metallidurans* AE126 and the zinc-resistant derivative AE126^R1^ were amplified by PCR (Phusion High-Fidelity DNA polymerase) with primer pairs CnrIns_Fw-CnrIns_Rv and CnrIns_Fw-CnrT_Rv (Supplementary Table 1), respectively, providing *Sac*I/*Bcu*I restriction sites. Afterwards, these PCR products were cloned as a *Sac*I/*Bcu*I fragment into pBBR1MCS2. The resulting pBBR1MCS2-*cnrYXH*, pBBR1MCS2-*cnrYXH*^R^, pBBR1MCS2-*cnrYXHCBAT*, and pBBR1MCS2-*cnrYXHCBAT*^R^ plasmids from *E. coli* DG1 transformants selected on LB Km^50^ were further confirmed by sequencing prior to transformation to *C*. *metallidurans* AE104. In addition, the *cnr* locus (*cnrYX*Δ*H::tetCBAT*) from *C*. *metallidurans* AE126 Δ*cnrH::tet* (see above) and the zinc-resistant derivative AE126 Δ*cnrH::tet*^R1^ (Table 1) were amplified by PCR (Phusion High-Fidelity DNA polymerase) with primer pair Cnr_Fw-RvpBBR1MCS2 (Supplementary Table 1). At the same time, the pBBR1MCS2 (Table 1) plasmid was linearized by PCR (Phusion High-Fidelity DNA polymerase) with primer pair pBBR1MCS2_Fw-Rv (Supplementary Table 1), providing homologous ends with the former amplified *cnr* locus. Afterwards, these PCR products were cloned using the GeneArt® Seamless Cloning and Assembly Enzyme Mix (Thermo Scientific) and the resulting pBBR1MCS2-*cnrYX*Δ*H::tetCBAT* and pBBR1MCS2-*cnrYX*Δ*H::tetCBAT*^R^ plasmids from a *E. coli* DG1 transformant selected on LB Km^50^ were further confirmed by sequencing prior to conjugation (triparental with *E. coli* HB101 pRK600 as helper) to *C*. *metallidurans* AE104.

### Monitoring of transcription

For quantitatively assaying endogenous promoter activity, *C*. *metallidurans* AE126 carrying luminescence reporter constructs (Table 1) were constructed to analyze expression from the IS*Rme5*, IS*1088*, and IS*1087B* transposase promoters. DNA fragments comprising the IS endogenous promoter (from the left inverted repeat until the transposase start codon) were amplified by PCR (Phusion High-Fidelity DNA polymerase) for IS*Rme5* (91 bp), IS*1088* (85 bp), and IS*1087B* (89 bp) with primer pairs, pIS*Rme5_*Fw-Rv, pIS*1088_*Fw-Rv, and pIS*1087B_*Fw-Rv, respectively (Supplementary Table 1), providing *EcoR*I/*Xba*I restriction sites. Similarly, fragments comprised of the sequence upstream of the transposase start codon up to the adjacent gene sequence were amplified for all four IS*Rme5* copies [209 bp for IS*Rme5*a (Rmet_1251), 257 bp for IS*Rme5*b (Rmet_1280), 297 bp for IS*Rme5*c (Rmet_1301), and 239 bp for IS*Rme5*d (Rmet_4152)] with primer pairs pIS*Rme5*a/b/c/d*_*Fw and pIS*Rme5*_Rv, respectively (Supplementary Table 1). Afterwards, these PCR products were cloned as *EcoR*I/*Xba*I fragments into pGLR1. The resulting pGLR1-P_*IS*__*Rme*5∕__*a*∕*b*∕*c*∕*d*_ -*gfpluxCDABE*, pGLR1-P_*IS*__1088_-*gfpluxCDABE*, and pGLR1-P_*IS*__1087*B*_-*gfpluxCDABE* plasmids from transformants selected on LB Km^50^ were further confirmed by sequencing prior to conjugation (triparental with *E. coli* HB101 pRK600 as helper) to *C*. *metallidurans* AE126 (Table 1). The luminescence reporter constructs were inoculated (in triplicate) into 4 ml of MM284 Km^1500^ and grown for 48 h. These stationary phase cells were diluted 1:100 (v/v) into fresh MM284 Km^1500^ and grown for 4 h. Next, exponential phase cell suspensions were divided and supplemented with different concentrations of Zn^2+^, Cd^2+^, Ni^2+^ or Co^2+^. For each sample, 200 μL was added to a 96-well black cell culture plate (Nunc Flat-bottom, Thermo Scientific) which was placed into a CLARIOstar® (BMG LABTECH). Plates were incubated at 30°C with shaking for several hours. At 30-min time intervals, the optical density at 600 nm and luminescence were measured. Data are shown as the ratio of the relative light unit to optical density (RLU/OD_600_) normalized to the MM284 control.

The expression of *cnrCBAT* from two zinc-resistant derivatives, AE126 Δ*cnrH::tet*^R1^ and AE126 Δ*cnrH::tet*^R2^ (Table 1), was quantitatively assayed via a transcriptional reporter fusion. A 953 and 853-bp DNA fragment comprising the *cnrC* promoter of AE126 Δ*cnrH::tet*^R1^ (harboring also a 3′ fragment of IS*Rme5* without its transposase promoter) and AE126 Δ*cnrH::tet*^R2^ harboring also a 3′ fragment of IS*1086* without its transposase promoter) were amplified by PCR (Phusion High-Fidelity DNA polymerase) with primer pairs IS*Rme5*_Internal-pCnrC_Rv and IS*1086*_Internal-pCnrC_Rv, respectively (Supplementary Table 1) In addition, as control, the native *cnrC* promoter of AE126 was amplified by PCR (Phusion High-Fidelity DNA polymerase) with primer pair pCnrC_Fw-Rv (Supplementary Table 1). At the same time, the pGLR1 (Table 1) plasmid was linearized by PCR (Phusion High-Fidelity DNA polymerase) with primer pair pGLR1_Fw-Rv (Supplementary Table 1), providing homologous ends with the former amplified fragments. Afterwards, these PCR products were cloned using the GeneArt® Seamless Cloning and Assembly Enzyme Mix (Thermo Scientific) and the resulting pGLR1-P_*IS*__*Rme*5*cnrC*_-*gfpluxCDABE*, pGLR1-P_*IS*__1086*cnrC*_-*gfpluxCDABE*, and pGLR1-P_*cnrC*_-*gfpluxCDABE* plasmids from an *E. coli* DG1 transformant selected on LB Km^50^ was further confirmed by sequencing prior to conjugation (triparental with *E. coli* HB101 pRK600 as helper) to *C*. *metallidurans* AE104 (Table 1). The luminescence reporter constructs were inoculated (in triplicate) into 4 ml of MM284 Km^1500^ and grown for 48 h. These stationary phase cells were diluted 1:100 (v/v) into fresh MM284 Km^1500^ and grown for 4 h. Next, 200 μL exponential phase cell suspensions were added to a 96-well black cell culture plate (Nunc Flat-bottom, Thermo Scientific) which was placed into a CLARIOstar® (BMG LABTECH). Plates were incubated at 30°C and the optical density at 600 nm and luminescence were measured.

## Results

### Isolation and phenotypic characterization of zinc-resistant AE126 derivatives

The minimal inhibitory concentration (MIC) of Zn^2+^ for *C. metallidurans* AE126, which is type strain CH34 cured from its pMOL30 mega plasmid encoding the *czc* operon (cadmium/zinc/cobalt resistance), is much less than its parent, being 0.2 and 12 mM, respectively. However, AE126 has the capability to spontaneously acquire additional zinc resistance, i.e., the ability to grow in the presence of 0.8 mM Zn^2+^ at a frequency of ~10^−6^ (Table 2; Collard et al., 1993). Interestingly, using a zinc plate assay two populations of zinc-resistant AE126 derivatives were observed that appeared at different incubation times. The first population, which arose after 2 days of incubation, showed a high variance-to-mean ratio (26 CFU) in a fluctuation assay with 20 independent cultures and a calculated mutation rate to zinc resistance of 0.105 ± 0.022 × 10^−7^ per generation. However, new colonies emerged on the selective plate in the ensuing days and we defined derivatives specifically emerging at day 5 as the second population. A higher mutation rate to zinc resistance of 0.307 ± 0.056 × 10^−7^ per generation and lower variance-to-mean ratio (3 CFU) was calculated for the second population. The lower mutation rate and high variance-to-mean ratio of the first population, with most plates yielding a variable number of mutant colonies while occasional plates showed a large number of mutants (so-called “jackpots”), suggested that the first population was pre-existing while the second population emerged on the plate itself.

**Table 2:** **Phenotypic characterization of ***C***. ***metallidurans*** strains carrying different parts of the ***cnr*** operon and their relation with resistance to different heavy metals**.

**Strains**	**Presence of**			**Cell survival frequency**		**MIC (mM)**		
	**CnrYX**	**CnrH**	**CnrCBAT**	**2d**	**5d**	**MIC Zn^2+^**	**MIC Ni^2+^**	**MIC Co^2+^**
AE104	−	−	−	0	0	0.2	0.3	0.3
AE126	+	+	+	10^−6^	10^−6^	0.2	2.5	5
AE126 Δ*cnrH::tet*	+	−	+	0	10^−8^	0.2	0.3	0.3
CH34 (*czc* operon)	+	+	+	1	1	12	2.5	5
AE126^R1^, AE126^R2^, AE126^R3^, AE126^R4^	−	+	+	1	1	2	9	12
AE126 Δ*cnrH::tet*^R1^, AE126 Δ*cnrH::tet*^R2^	+	−	+	1	1	2	9	12
AE104 pBBR1MCS2	−	−	−	0	0	0.2	0.3	0.3
AE104 pBBR1MCS2-*cnrYXH*	+	+	−	0	0	0.2	0.3	0.3
AE104 pBBR1MCS2-*cnrYXH*^R^	−	+	−	0	0	0.2	0.3	0.3
AE104 pBBR1MCS2-*cnrYXHCBAT*	+	+	+	10^−6^	10^−6^	0.2	2.5	5
AE104 pBBR1MCS2-*cnrYXHCBAT*^R^	−	+	+	1	1	2	9	12
AE104 pBBR1MCS2-*cnrYXΔH::tetCBAT*	+	−	+	0	10^−8^	0.2	0.3	0.3
AE104 pBBR1MCS2-*cnrYXΔH::tetCBAT*^R^	+	−	+	1	1	2	9	12

*Cell survival frequency after exposure to 0.8 mM Zn^2+^ was determined after 2 and 5 days of incubation, respectively. Minimum inhibitory concentrations (MICs) of different heavy metals were determined after 2 days of incubation*.

Cultured separately in liquid MM284 without zinc demonstrated similar growth rates for AE126 and zinc-resistant derivatives from the first (AE126^R1^) and second (AE126^R3^) population (Table 3). Moreover, the zinc-resistant derivatives exhibited a similar lag phase that was shorter than that of AE126 (Table 3). In addition, both derivatives grew equally fast in the presence of 0.8 mM Zn^2+^ but displayed a prolonged lag phase compared to MM284 without zinc (Table 3). Next to increased Zn^2+^ resistance (a 10-fold increase of the MIC), the characterized zinc-resistant AE126 derivatives also displayed an increased resistance to Ni^2+^, Co^2+^ and Cd^2+^ (3, 2.5 and two-fold increase of the MIC, respectively). However, the level of zinc and cadmium resistance of these mutants is still considerably lower than those from the type strain *C*. *metallidurans* CH34 (Table 2).

**Table 3:** **Growth parameters of different ***C***. ***metallidurans*** strains in MM284 medium with and without 0.8 mM Zn^**2+**^**.

***C*. *metallidurans***	**MM284**		**MM284 0.8 mM Zn**^**2+**^	
	**Lag phase (h)**	**Growth rate (1/h)**	**Lag phase (h)**	**Growth rate (1/h)**
AE126	22.02 ± 1.19	0.04 ± 0.01	No growth	
AE126^R1^	17.62 ± 0.35^*,$^	0.05 ± 0.00	29.54 ± 2.66^*,$^	0.11 ± 0.07^*,$^
AE126^R3^	18.32 ± 0.32^*,$^	0.05 ± 0.00	26.00 ± 3.18^*,$^	0.09 ± 0.06^*,$^
AE126 Δ*cnrH::tet*	23.27 ± 2.79	0.06 ± 0.02	No growth	
AE126 Δ*cnrH::tet*^R1^	18.65 ± 0.27^*,$^	0.05 ± 0.00	28.91 ± 3.11^*,$^	0.11 ± 0.09^*,$^

Parameter values are means ± standard deviations. Strains for which the estimates are significantly different (based on ANOVA and post-hoc Tukey) from AE126 and AE126 ΔcnrH::tet are marked with * and ^$^, respectively.

### Genetic characterization of zinc-resistant AE126 derivatives

**Figure 1:**
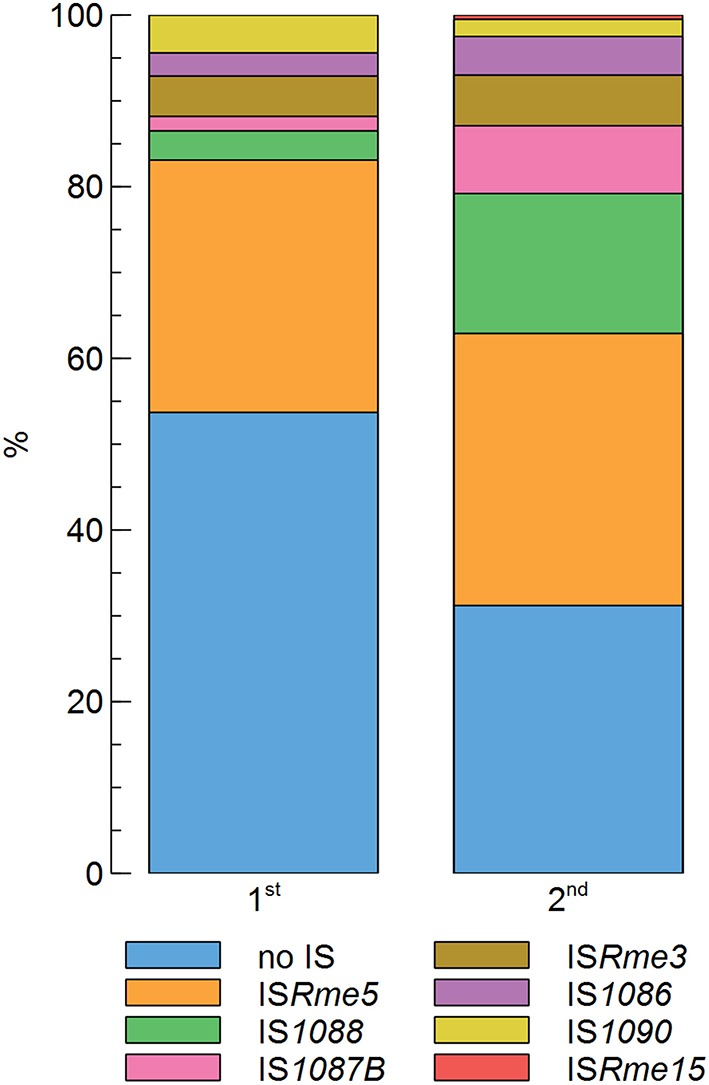
**Contribution of different types of ***cnrYX*** mutations in the first and second population of zinc-resistant ***C***. ***metallidurans*** AE126 derivatives comprising 296 and 202 mutants, respectively**.

In previous work, only two zinc-resistant AE126 derivatives were analyzed (Collard et al., 1993; Grass et al., 2000). Therefore, we scrutinized *cnrY* and *cnrX* of 498 randomly selected zinc-resistant AE126 isolates, of which 296 belong to the first population and 202 belong to the second population, by PCR and sequencing to determine if *cnrY* and *cnrX* were indeed the sole targets leading to increased zinc resistance of AE126 derivatives (Supplementary Figure 1). This analysis confirmed that all 498 randomly selected isolates either had an IS element inserted or another mutation (point mutation, small insertions, or deletion) in *cnrY* or *cnrX* (Figure 1). Interestingly, however, the first population (46.3%) had a significantly lower contribution of IS elements than the second population (68.8%) as determined by a Fisher Exact test with a *p* < 0.05 (Figure 1), indicating that transposition of these IS elements might be promoted by the selective zinc condition.

### Characterization of IS-independent zinc-resistant AE126 mutants

Regarding the IS-independent mutations in *cnrYX*, it is clear that only mutations resulting in inactivation of either CnrY or CnrX are selected in the zinc plate assays. The IS-independent mutations are dispersed throughout the *cnrYX* locus and include frameshift mutations, non-sense mutations and larger deletions. For instance, a 360-nucleotide deletion was observed including the complete *cnrY* gene.

Two zinc-resistant IS-independent AE126 derivatives, AE126^R2^ (harboring a frameshift mutation caused by a single nucleotide deletion in *cnrX*) and AE126^R3^ (harboring a five nucleotide 5′-CGCGA-3′ duplication in *cnrY*), both belonging to the second population of zinc-resistant AE126 derivatives, were selected for whole-genome expression profiling to further confirm the *cnr*-dependency underlying toxic zinc stress and subsequent acquired zinc resistance. Although, IS-independent mutations are discussed here, the five nucleotide duplication in *cnrY* of AE126^R3^ might suggest an insertion and subsequent excision event of an IS element resulting in a direct target repeat in *cnrY*. Both AE126^R2^ and AE126^R3^ bear mutations leading to inactivation of the anti-sigma CnrYX complex, and whole-genome expression profiling showed 219 and 86 open reading frames (ORFs) that were significantly differentially expressed (>2-fold with an adjusted *p* < 0.05) in comparison with the parental AE126 strain, respectively (Supplementary Table 2). Twenty ORFs were commonly differentially expressed in both zinc-resistant derivatives. The mutated regulatory genes *cnrY* and *cnrX* were the most upregulated (11- and 15-fold in AE126^R2^, and 33- and 57-fold in AE126^R3^, respectively) followed by the structural locus *cnrCBAT* (nine-fold average for AE126^R2^ and 18-fold average for AE126^R3^). Transcription of *cnrH* was three- or six-fold upregulated in AE126^R2^ and AE126^R3^, respectively (Supplementary Table 2). As such, it seems that constitutive overexpression of the *cnr* pump is responsible for the acquired zinc resistance. The high amount of other differentially expressed genes and the fact that most of these genes (93%) were unique for one of both zinc-resistant derivatives indicate that probably different mutations, not involved in the acquired zinc resistance, occurred during the selective exposure to Zn^2+^, which thus may comprise a more general response to stress with mutations randomly happening, supporting zinc-induced mutagenesis for the second population of zinc-resistant derivatives.

To further and unambiguously confirm the essential role of the *cnrYXHCBAT* locus in the capability to spontaneously acquire zinc resistance, complementation assays were performed in the zinc-sensitive and plasmidless *C. metallidurans* CH34 derivative, i.e., strain AE104 (Table 2). The findings of Collard et al. (1993) that strain AE104 can never be evolved to withstand the toxic zinc concentration used in zinc plate assays (0.8 mM Zn^2+^) was confirmed. Complementation of *C*. *metallidurans* AE104 with the complete *cnr* locus, thus both the regulatory and structural genes, resulted in the ability to acquire zinc resistance (frequency of 0.96 ± 0.38 × 10^−6^ after 48 h exposure to 0.8 mM Zn^2+^), whereas complementation of AE104 with the complete but modified *cnr* locus from AE126^R1^ resulted in full resistance (frequency of 100%; Table 1). To test if the CnrH sigma factor could drive other potential resistance genes as well, the parental regulatory *cnrYXH* locus, and the mutated *cnrYXH* locus from AE126^R1^ were introduced in AE104 but no zinc resistance could be acquired. Thus, the presence of the *cnrCBAT* genes encoding the RND-driven efflux system was essential to acquire zinc resistance (Table 2). These complementation assays showed that the *cnrYXHCBAT* cluster is necessary for acquiring zinc resistance at high frequency in *C*. *metallidurans* AE104 or AE126 and that the inactivation of *cnrYX* and concomitant CnrH-mediated derepression of *cnrCBAT* is responsible for this zinc resistance.

### Characterization of IS-dependent zinc-resistant AE126 mutants

**Figure 2:**
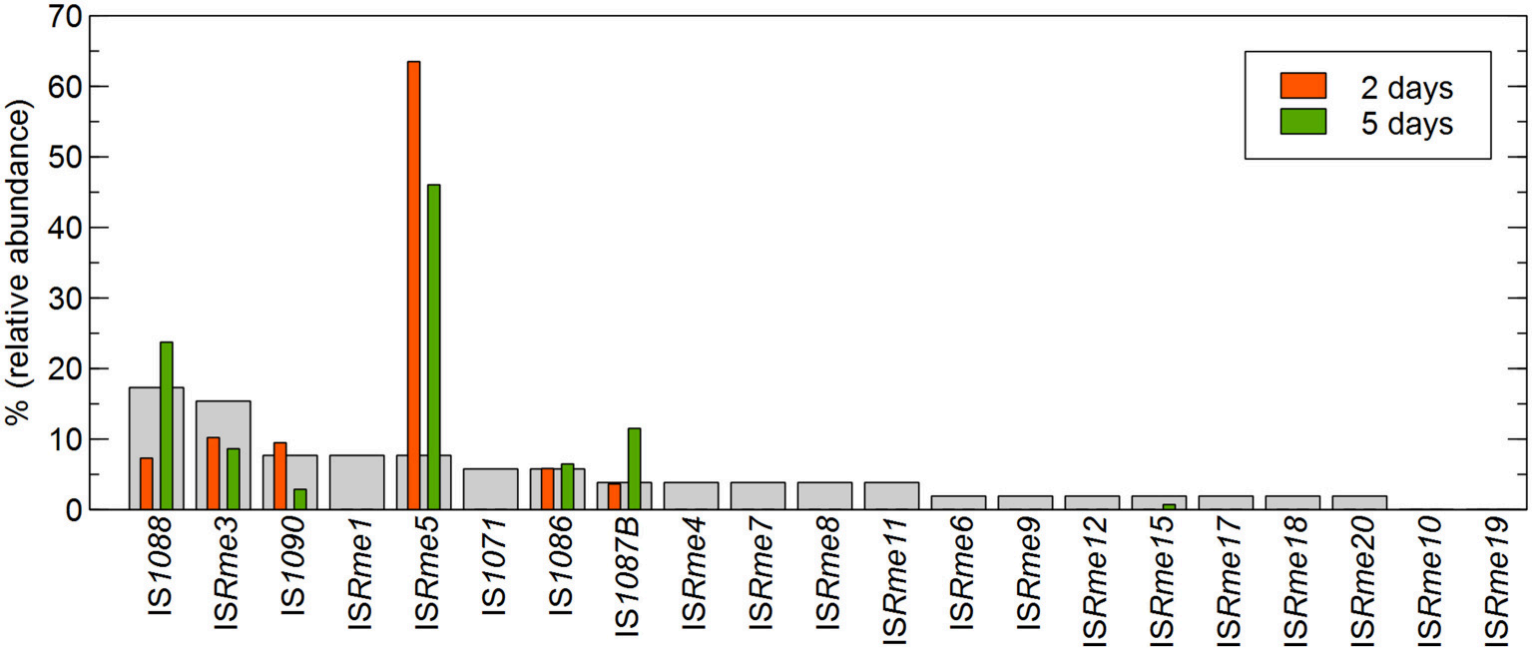
**Correlation between the copy number and transposition of IS elements in the ***cnrYX*** locus identified in zinc-resistant AE126 derivatives**. Red and green bars represent the relative abundance of a specific IS element identified in the pool of IS-mediated zinc-resistant AE126 derivatives isolated after 2 and 5 days of incubation, respectively. Light gray bars represent the relative abundance of a specific IS element in the genome of *C*. *metallidurans* AE126.

Focusing further on the IS-dependent zinc-resistant mutants, IS*Rme5* appeared to be the main contributor to the obtained insertions both in the first (29.4%) and second (31.7%) population (Figure 1). Other identified IS elements included IS*Rme3*, IS*Rme15*, IS*1090*, IS*1088*, IS*1086* and IS*1087B*. Comparing the contribution of specific IS elements in the zinc-resistant AE126 population to their abundance in the genome showed that some IS elements were clearly more represented than would be expected from their copy number (Figure 2). IS*Rme5* (four identical copies in the genome) had a significant higher contribution to both populations, and IS*1087B* (two identical copies in the genome) to the second population as determined by a Fisher Exact test with a *p* < 0.05. In addition, IS*1088* (nine identical copies in the genome) was significantly more represented in the second than in the first population (Fisher Exact test; *p* < 0.05), but comparing its contribution to the second population to its abundance in the genome revealed no significant higher contribution than would be expected from its copy number (Figure 2).

**Figure 3:**
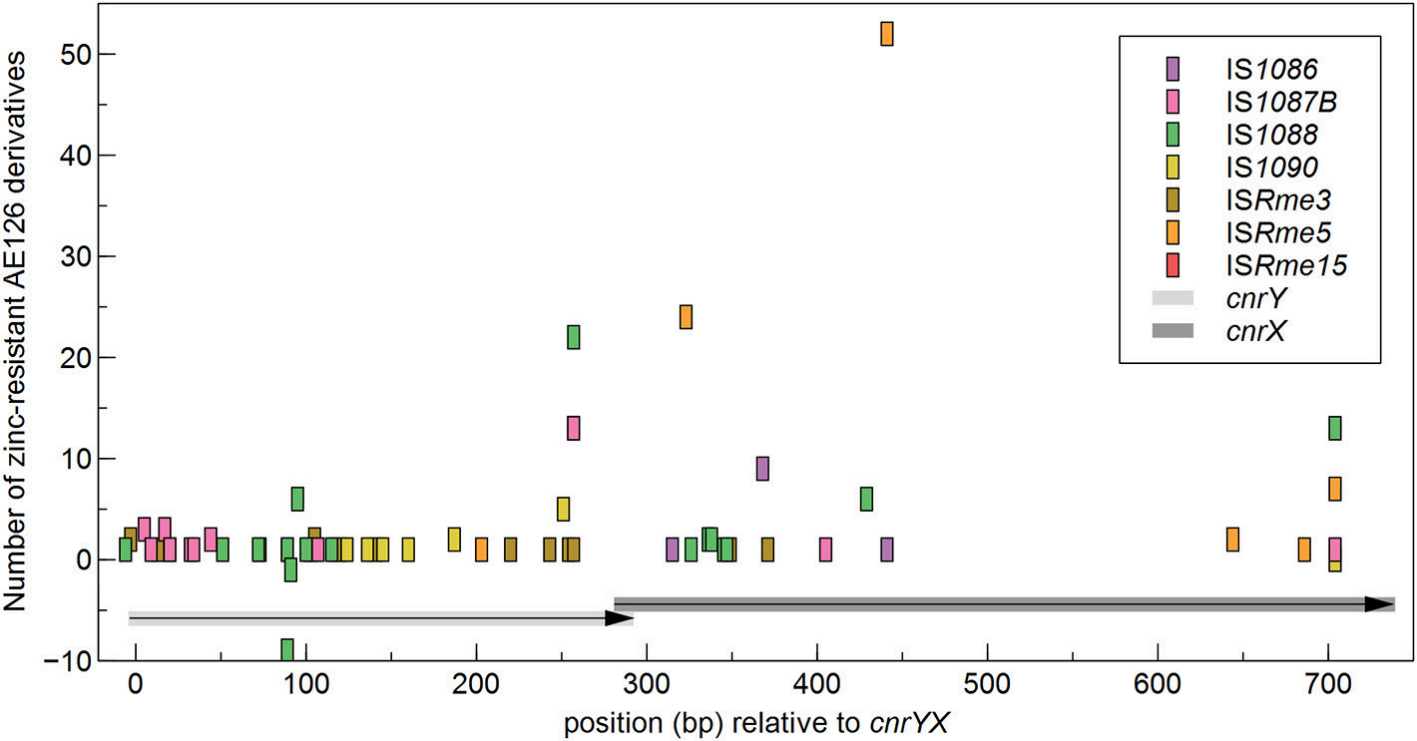
**Distribution of IS elements in the ***cnrYX*** locus of zinc-resistant AE126 derivatives**. Insertions in the same and opposite transcriptional orientation as the *cnr* operon are shown as positive and negative values, respectively.

Next, the identified IS insertion sites were scrutinized. A different use of target genes (i.e., *cnrY* or *cnrX*) was detected for the identified IS elements, although this had no influence on zinc resistance as determined by cell survival at 0.8 mM Zn^2+^ and by MIC testing (data not shown). Most IS elements inactivated *cnrY* with the exception of IS*Rme5* and IS*1086*. In general, all IS elements, except IS*1088*, inserted exclusively in the same transcriptional orientation as the disrupted gene. No difference in target site use was detected between mutants from the first or second population. IS*Rme5* used six different insertion sites throughout the target locus and two were used preferentially (Figure 3).

**Figure 4:**
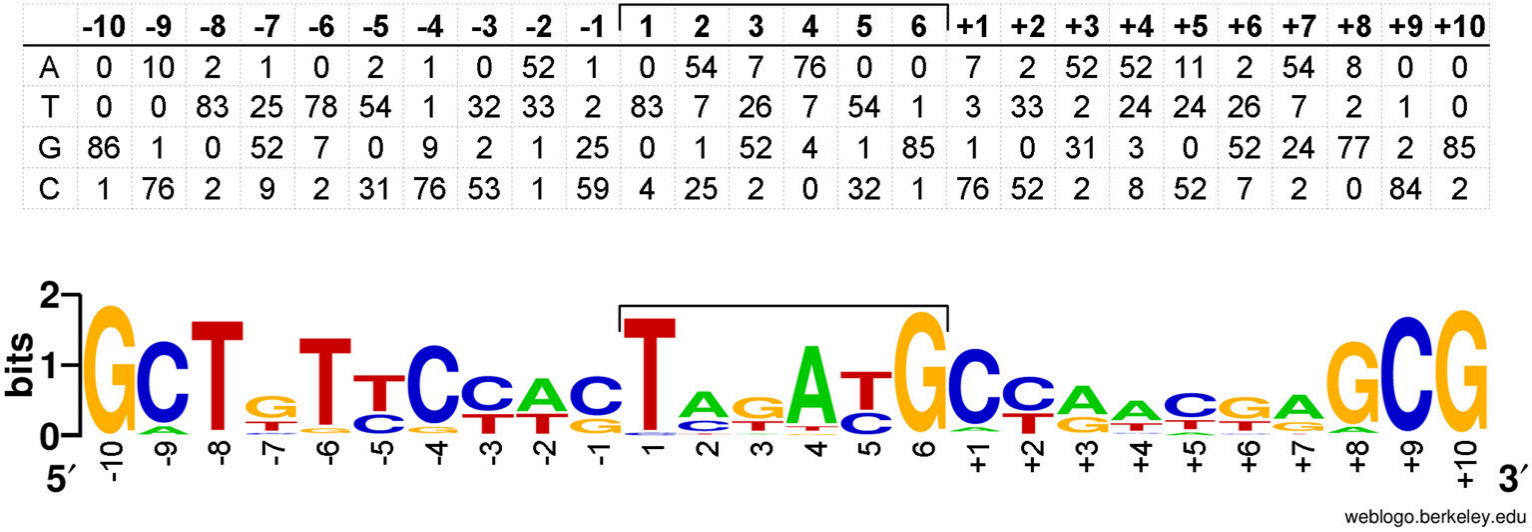
**Position-specific scoring matrix derived from the multiple sequence alignment of all target sites used by IS***Rme5*****. Eighty-seven target sites and the flanking DNA sequences have been aligned to generate the matrix shown. The sequences compiled are those adjacent to the left (−1 to −10) and right end (+1 to +10) of IS*Rme5*. The central six nucleotides are those that would be duplicated on insertion. A sequence logo was generated from this matrix with WebLogo (Crooks et al., 2004).

A position-specific scoring matrix was derived from the multiple sequence alignment of all IS*Rme5* target sites (Figure 4). This position-specific scoring matrix was used to scan the genome sequence of *C*. *metallidurans* CH34 via the RSAT motif scan (Turatsinze et al., 2008) and resulted in numerous hits (Supplementary Table 3). Besides *cnrX*, other genes involved in regulation are found in the list composed of the top 100 hits, including genes encoding for transcriptional regulators, receptor proteins, a sensor histidine kinase, and a signal transduction protein. In addition, the family of transporters is with 21 genes also overrepresented in the motif scan. The other identified IS elements in *cnrYX* showed a more random insertion pattern although IS*1087B* preferentially inserted into the 5′-region of the regulatory locus.

### Characterization of cnrH-independent zinc-resistant AE126 mutants

To further analyze the adaptation capacities of *C*. *metallidurans* AE126 to toxic zinc concentrations, a *cnrH* deletion strain was created to assess if other resistance mechanisms than CnrH-mediated expression of *cnrCBAT* could result in increased zinc resistance. No difference in zinc resistance was observed between the parental and Δ*cnrH* deletion strain, however, as expected, the *cnrH* deletion resulted in a drastically decreased cobalt and nickel resistance (Table 2). Interestingly, zinc-resistant AE126 Δ*cnrH::tet* derivatives could be isolated after exposure to 0.8 mM Zn^2+^, but only after a minimum exposure time of 96 h and at a frequency 100-fold less compared with AE126 (Table 2). Cultured separately in liquid MM284 without zinc demonstrated similar growth rates for AE126, AE126 Δ*cnrH::tet*, and the zinc-resistant derivative AE126 Δ*cnrH::tet*^R1^ (Table 3). In addition, again, the zinc-resistant derivate exhibited a lag phase that was shorter than that of AE126 Δ*cnrH::tet* and AE126 (Table 3). Moreover, AE126 Δ*cnrH::tet*^R1^ displayed the same growth rate in the presence of 0.8 mM Zn^2+^ than the CnrH-dependent zinc-resistant AE126^R1^ and AE126^R3^ (Table 3).

**Figure 5:**
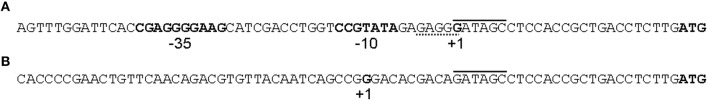
**(A)** Sequence of the intergenic region between *cnrH* and *cnrC* with the last three nucleotides (ATG) being the start codon of *cnrC*. The −35 and −10 box and transcription start of *cnrC* are highlighted in bold. Integration sites of IS*Rme5* (full line) and IS*1086* (dashed line) are indicated above and below the sequence, respectively. **(B)** Sequence of the newly formed *cnrC* promoter via IS*Rme5* insertion with direct repeat at the point of insertion (full line) and transcription start.

Phenotypic characterization of zinc-resistant AE126 Δ*cnrH::tet* derivatives further indicated an increased resistance to Ni^2+^ (22-fold) and Co^2+^ (32-fold; Table 2), indicative for a role of the *cnr* locus. Genetic characterization of six independent zinc-resistant AE126 Δ*cnrH::tet* derivatives revealed for five derivatives an insertion of IS*Rme5* at the same target site 25 bp upstream the *cnrC* start codon (Figure 5). One representative was further used and designated AE126 Δ*cnrH::tet*^R1^. The other derivative (AE126 Δ*cnrH::tet*^R2^) carried an IS*1086* insertion 29 bp upstream of the *cnrC* start codon (Figure 5). To exclude any other possible involvement of additional resistance determinants, complementation assays were performed where the complete but modified *cnr* operon of AE126 Δ*cnrH*::*tet* and AE126 Δ*cnrH*::*tet*^R1^ were transferred to *C*. *metallidurans* AE104, respectively. These complementation assays demonstrated that *cnrYX*Δ*cnrH*::*tetCBAT*^R^ mediated the increased zinc resistance (Table 2). Furthermore, transcriptional reporter fusions with *cnrC* promoter fragments of AE126 Δ*cnrH::tet*^R1^ (953 bp fragment harboring also a 3′ fragment of IS*Rme5* without its transposase promoter) and AE126 Δ*cnrH::tet*^R2^ (853 bp fragment harboring also a 3′ fragment of IS*1086* without its transposase promoter) confirmed the constitutive expression of *cnrCBAT* (Supplementary Figure 2). Therefore, the insertion of these IS elements in the intergenic region between Δ*cnrH::tet* and *cnrC* resulted in a constitutively increased transcription of *cnrCBAT*, which was also confirmed by microarray analysis of AE126 Δ*cnrH::tet*^R1^ (Supplementary Table 4). Since read-through expression from the transposase promoter did not account for this increased expression, the transcription start site of *cnrCBAT* in AE126 Δ*cnrH::tet*^R1^ (IS*Rme5* insertion) was identified and showed that expression is driven by an outward-directed promoter positioned at the 3′ extremity of IS*Rme5* (Figure 5). This could also be deduced for IS*1086* based on the insertion site relative to the transcription start site of *cnrC* (Figure 5).

### Endogenous promoter activity of IS elements

**Figure 6:**
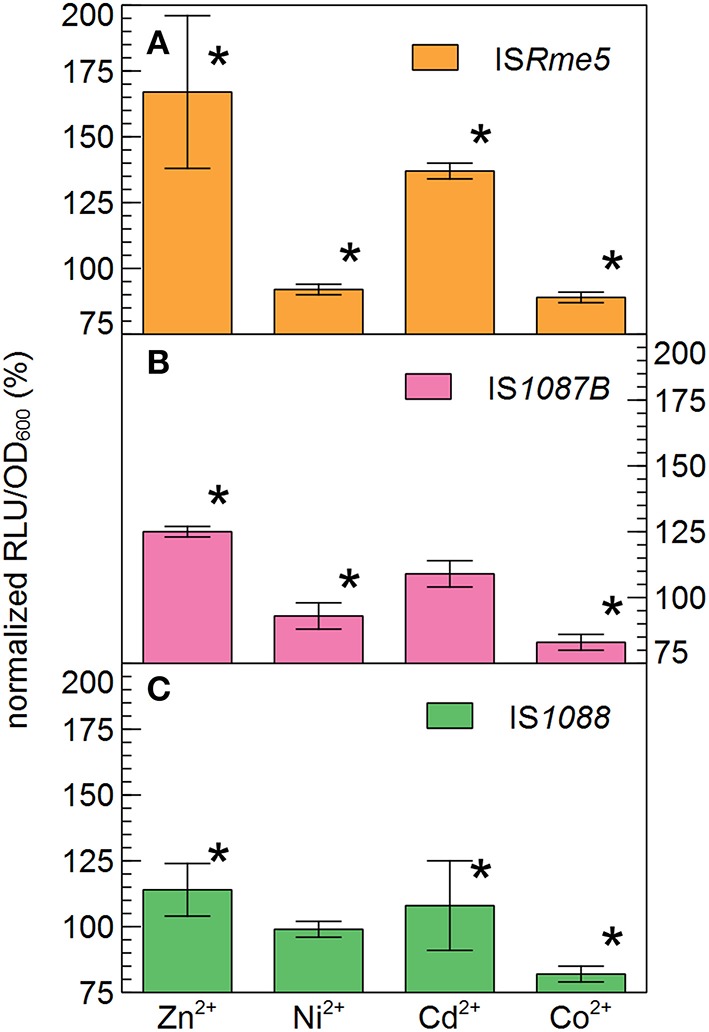
**Endogenous promoter activity of the IS***Rme5*** (A), IS***1088*** (C), and IS***1087B*** (B) transposase in response to Zn^**2+**^ (100 μM), Ni^**2+**^ (100 μM), Cd^**2+**^ (10 μM) and Co^**2+**^ (50 μM) after 4 h of incubation, calculated by normalizing the reporter signal RLU to cell density ratio (RLU/OD_**600**_) to that of the control without added metals**. ^*^indicates a significant difference between the tested metal ion and the control, determined by a *t*-test with a *p* < 0.05. The average values of three independent experiments with standard deviations are shown.

**Figure 7:**
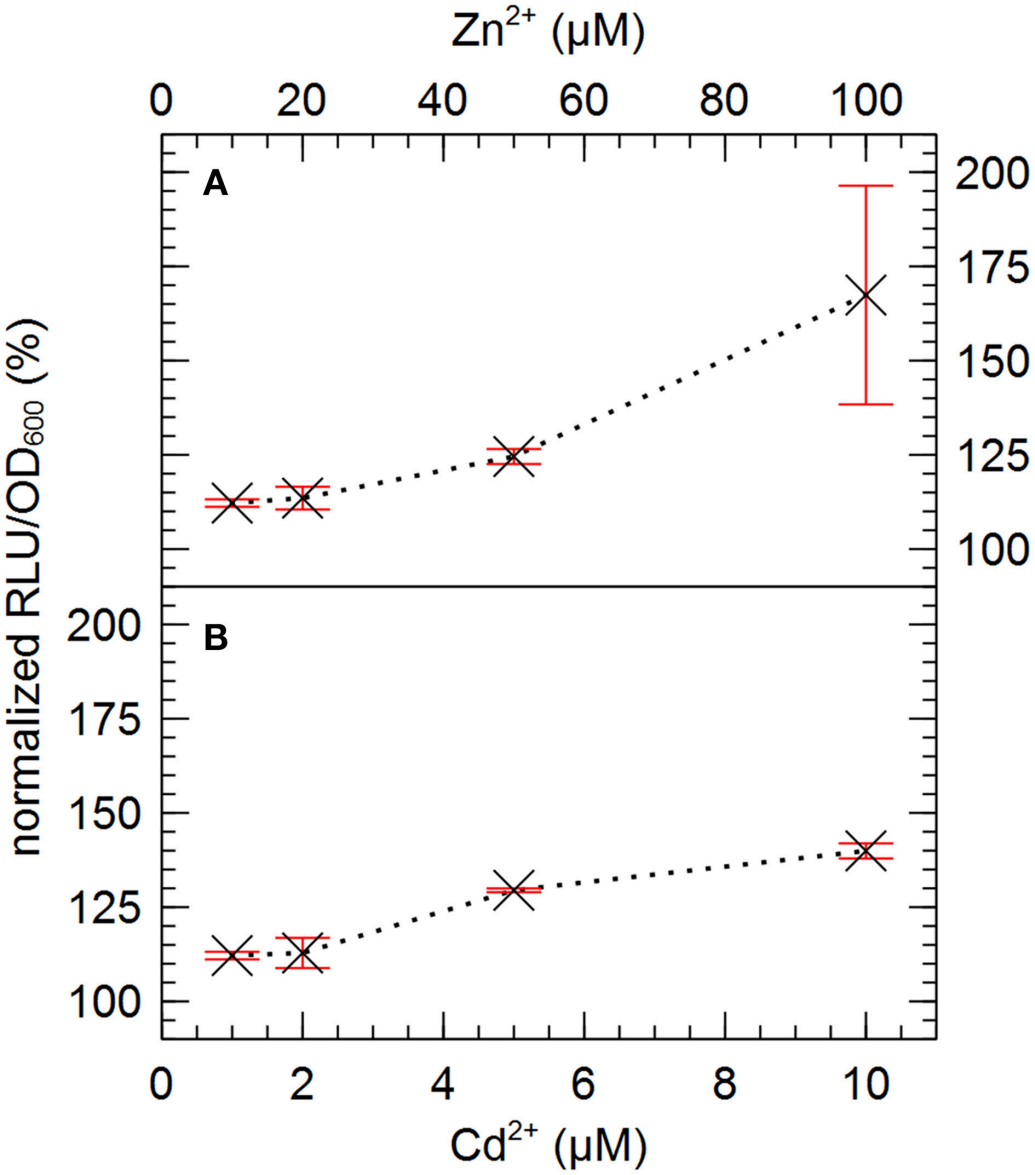
**Normalized dose-response curve for the transcription of the endogenous promoter of IS***Rme5*** in response to zinc (A) and cadmium (B) measured after 4 h of incubation**. The average values of three independent experiments with standard deviations are shown.

Based on their relative high abundance in the zinc-resistant derivatives of AE126, especially in the second population, IS*Rme5*, IS*1088* and IS*1087B* were selected for further investigation. To analyze the effect of metal ion stress on the endogenous promoter activity of the transposase of the three selected IS elements, transcriptional fusions between the promoterless tandem bi-cistronic *gfp*-*luxCDABE* reporter and fragments of IS*Rme5*, IS*1088*, and IS*1087B* comprising the left inverted repeat up until the start codon of the transposase were constructed and analyzed. The promoters of all three IS elements were induced by Zn^2+^ and Cd^2+^, but not by Ni^2+^ and Co^2+^ (Figure 6). Of the three endogenous promoters analyzed, the IS*Rme5* promoter was induced the most by Zn^2+^, and in lesser extent by Cd^2+^ (1.7-fold for Zn^2+^ and 1.4-fold for Cd^2+^ after 4 h). A dose-dependent response was observed with the highest response for 100 μM Zn^2+^ and 10 μM Cd^2+^, respectively (Figure 7). Higher doses of Zn^2+^ or Cd^2+^ could not be tested as they are detrimental to the growth of *C*. *metallidurans* AE126 strains. The IS*1088* and IS*1087B* endogenous transposase promoters were induced 1.14 and 1.25-fold by Zn^2+^ (after 4 h), respectively, and to a lesser extent by Cd^2+^.

### Analysis of upstream flanking genomic sequence on IS*Rme5* transposase expression

**Figure 8:**
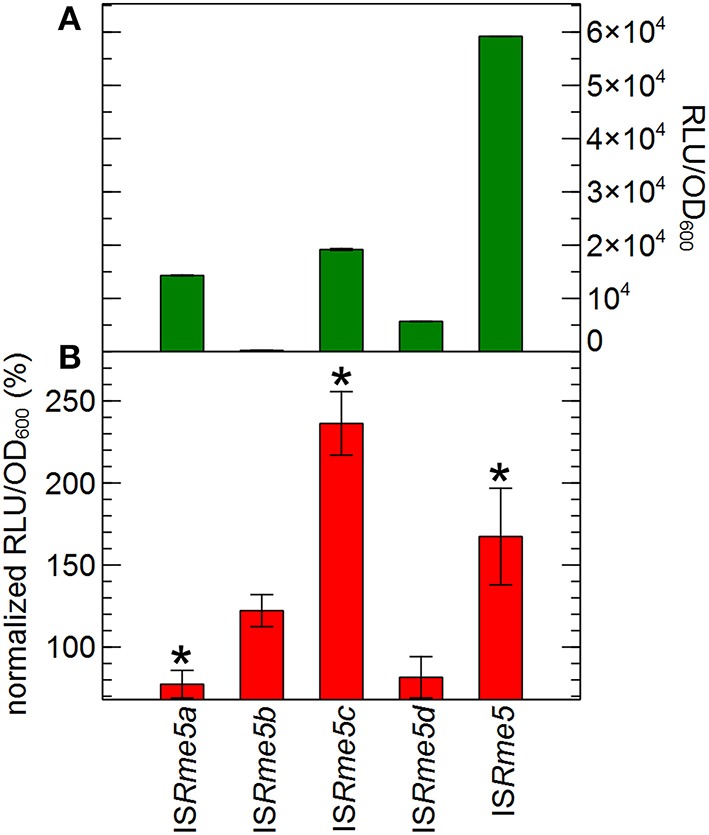
**Transposase expression of the different IS***Rme5 copies*****. Reporter constructs contain the IS*Rme5* tranpsosase promoter (starting from the left inverted repeat) and for the four IS*Rme5* (a, b, c, and d) copies, in addition, the DNA sequence (~250 bp) upstream the integration site. **(A)** Basal expression of the reporter constructs at the start of the experiment, calculated by normalizing the reporter signal RLU to cell density (RLU/OD_600_). **(B)** Expression after 4 h of incubation in the presence of 100 μM Zn^2+^, calculated by normalizing the reporter signal RLU to cell density (RLU/OD_600_) to that of the control without added Zn^2+^. ^*^indicates significant difference between Zn^2+^ and control, determined by a *t*-test with a *p* < 0.05. The average values of three independent experiments with standard deviations are shown.

For IS*Rme5*, the most active element in response to zinc, the effect of the upstream flanking genomic sequence on transposase expression was scrutinized in more detail. *C*. *metallidurans* AE126 harbors three copies of the IS*Rme5* element on the chromosome and one copy on the chromid, which are identical at the sequence level. However, the transposase open reading frame contains no stop codon within the IS element, implying that the different IS*Rme5* transposases, encoded by the four copies, have different lengths generated by sequence specific insertion. This is not unique as more members of the IS*481*-family do not display a stop codon for the transposase open reading frame within their respective IS elements (Redenbach et al., 1996; Tauch et al., 1998). The transposase of the corresponding IS*Rme5*a, b, c and d element consist of 333, 463, 321 and 321 amino acids, respectively. Two copies are located in genomic island CMGI-2, which is involved in hydrogenotrophy and the metabolism of aromatic compounds (Van Houdt et al., 2009; Mijnendonckx et al., 2011). Transcriptional fusions between the promoterless tandem bi-cistronic *gfp-luxCDABE* reporter and sequences starting 118, 166, 206 or 148 bp upstream the left inverted repeat up until the transposase start codon of all four IS*Rme5* copies were constructed and analyzed. Comparing the basic expression activity, it is clear that the genomic sequence upstream the left inverted repeat of all IS*Rme5* copies exerts a negative effect on IS*Rme5* endogenous promoter activity. This is especially true for IS*Rme5*b where almost no expression is detected, i.e., < 0.5% (Figure 8). Furthermore, although the reporter construct composed of only the promoter region of IS*Rme5*, thus without additional genomic sequences, is Zn^2+^-inducible, no Zn^2+^ induction was detected for the reporter constructs composed of upstream genomic content of IS*Rme5*a, IS*Rme5*b and IS*Rme5*d. The reporter construct of IS*Rme5*c, however, was more than two-fold induced for Zn^2+^ (Figure 8).

### Enhanced adaptation potential to other stress challenges

The effect of IS transposition in the *cnrYX* genes on the general fitness was analyzed. In this test, the parental AE126 and the zinc-resistant *C*. *metallidurans* AE126^R4^ strain were used. AE126^R4^ carries an additional IS*Rme5* element inserted in *cnrX* in the same transcriptional orientation as the *cnr* operon, yielding a continuous read-through IS*Rme5* transposase expression as whole-genome expression verified that expression of *cnrYX* is induced in CnrH-dependent zinc-resistant AE126 derivatives (Supplementary Table 2). The pJV240 plasmid was introduced into AE126 and AE126^R4^, and biological triplicates were subsequently exposed to 10% sucrose. Sucrose-resistant derivatives were isolated and the pJV240-encoded *sacBR* locus was screened by PCR to determine the involvement of IS*Rme5*. AE126^R4^ pJV240 displayed a higher survival frequency (4.78 ± 1.58 × 10^−6^) than AE126 pJV240 (1.42 ± 0.30 × 10^−7^) and IS*Rme5* was found to disrupt *sacBR* in sucrose-resistant AE126^R4^ pJV240 derivatives (3/20) opposed to sucrose-resistant AE126 pJV240 derivatives (0/20).

## Discussion

MGEs have been shown to provide their hosts with a wide diversity of adaptive traits. Although, transposition of IS elements mostly results in detrimental mutations, a fraction of insertions can be beneficial and lead to increased survival. The simplest effect of IS transposition is the inactivation of a gene at a particular target site. In addition, IS transposition can also lead to altered expression of adjacently located genes by co-transcription or by providing a (hybrid) promoter or other protein binding elements located inside the IS element.

It was previously demonstrated that *C*. *metallidurans* AE126 could readily acquire increased zinc resistance (Collard et al., 1993). We observed that zinc-resistant AE126 derivatives arose at different time points after exposure to 0.8 mM Zn^2+^ and all 498 tested derivatives had mutations targeting the regulatory locus of the RND-driven CnrCBA efflux system. The first population arose after an exposure time of 48 h and new derivatives kept appearing in the ensuing days with a second population defined as derivatives that arose after an exposure time of 120 h. The first population displayed a high variance-to-mean ratio, with most plates yielding a variable number of mutant colonies while occasional plates showed a large number of mutants (so-called “jackpots”), indicating the involvement of pre-existing mutations (Luria and Delbrück, 1943). Interestingly, the second population displayed a higher mutation rate and lower variance-to-mean ratio. Although, the latter was still three times the theoretical value of one when only stress-induced mutagenesis is considered, the much lower value and higher mutation rate are indicative for the involvement of zinc-induced mutagenesis in the second population. Furthermore, mutants from both populations have the same growth rate on zinc medium suggesting once more that the second population may originate from selection on the selective zinc plate. Adaptive mutation and accumulation of colonies throughout incubation time has been described for the activation of a promoterless phenol degradation operon in starving *Pseudomonas putida*. Although, phenol-utilizing mutants already appeared on phenol-minimal plates on day 2, the majority of the colonies began to appear on day 3 and continued to accumulate during the next 7 days (Kasak et al., 1997). In addition, mutations to ciprofloxacin resistance also continually occurred in non-dividing *E. coli* cells during a 7-day exposure to ciprofloxacin in agar and the late-appearing resistant mutants had no growth rate defect compared to wild type or early-appearing mutants (Riesenfeld et al., 1997). Moreover, *E. coli* cells could utilize glycerol in the absence of Crp by activation of the silent *glpFK* glycerol utilization operon by transposition of IS*5* upstream of *glpFK*. The transposed IS*5* element harbors a permanent bend and IHF binding site at its 3′-end that promoted the expression of the adjacently located *glpFK* operon (Zhang and Saier, 2011). When *crp*^−^ cells of *E*. *coli* are incubated on solid glycerol minimal medium, Glp^+^ colonies first appeared after 3 days of incubation and new colonies continued to appear in the ensuing days. However, wild type and *crp* Glp^+^ cells plated on the same solid medium formed already colonies in less than 2 days, indicating that the Glp^+^ mutants that arose from the *crp* cells on glycerol minimal medium plates were not present in the cell culture before plating (Zhang and Saier, 2011).

For acquiring zinc resistance at high frequency (10^−6^), inactivation of anti-sigma factor CnrYX complex is essential to liberate CnrH and increase *cnrCBAT* expression. Although the efflux pump CnrCBAT is necessary to mediate zinc efflux, the role of the sigma factor CnrH in promoting *cnrCBAT* expression is not. In the absence of *cnrH*, expression of *cnrCBAT* and acquisition of zinc resistance could be mediated by the correct insertion of IS*Rme5* or IS*1086* carrying an outward-directed promoter positioned at their 3′ extremity, albeit to a lower frequency (10^−8^), and only after a minimum exposure time of 96 h. These mutants displayed the same growth rate on zinc medium than CnrH-dependent zinc-resistant AE126 derivatives from the first population, which were isolated after 48 h, suggesting that these CnrH-independent zinc-resistant derivatives may originate from selection on the selective zinc plate. In addition, using the cRACE method, the wild type promoter transcription start site of the *cnrCBAT* structural resistance genes was found to be located downstream of *cnrH* as previously reported by Grass et al. (2000) and not upstream as previously reported by Tibazarwa et al. (2000).

Exposure of AE126 to 0.8 mM Zn^2+^ revealed transposition of seven different IS elements in the *cnrY* or *cnrX* gene (occurrence of transposition IS*Rme5* > IS*1088* > IS*Rme3* > IS*1087B* > IS*1090* > IS*1086* > IS*Rme15*). Active IS transposition in *C. metallidurans* was already observed for IS*Rme1*, IS*Rme3*, IS*1086*, IS*1087B*, IS*1088* and IS*1090* (Dong et al., 1992; Collard et al., 1993; Grass et al., 2000; Ma-e-Talat, 2000; Schneider et al., 2000b; Tibazarwa et al., 2000; Mijnendonckx et al., 2011), but not for IS*Rme5* and IS*Rme15*. Overall, IS elements contributed more to the second population that arose after five days of incubation on plate. IS*Rme5* was the main IS contributing to both populations, whereas IS*1088* and IS*1087B* showed a higher contribution to the second compared with the first population. Most of the transposed IS elements (>95%) inserted exclusively in the same transcriptional orientation as *cnrCBAT*, although inactivation of CnrYX is sufficient to acquire zinc resistance. It is unclear why a given site should be used in only one orientation (Lampe et al., 1998). Most transposases did not show insertion site specificity, only for IS*Rme5* two hotspot loci were identified that were used preferentially and specifically by IS*Rme5*. Scanning the genome of *C*. *metallidurans* CH34 with the generated target sequence matrix resulted in numerous hits, with various hits in genes involved in regulation and transport processes, indicating the evolutionary power that IS*Rme5* might exert as inactivation of those genes mostly results in significant phenotypic alterations. In some cases transposases prefer a particular DNA structure, most notably bent DNA, as target site (Craig, 1997). It seems that this was the case for the IS*1087B* transposase as it mostly inserted in the 5′-region of *cnrY*, indicating that the IS*1087B* transposase does not recognize target DNA in any sequence-specific manner but that certain sequences surrounding the insertion site may allow local structures to form that are conductive for IS*1087B* insertion. There is precedence for this phenomenon in the non-random use of target sequences of P transposable elements, where the local target preference depends more on DNA structure than on primary sequence (Liao et al., 2000).

Our study showed for the first time that the endogenous promoters of the IS*Rme5*, IS*1088* and IS*1087B* transposases can be induced by metal ions. This increased promoter induction was specific for zinc and cadmium but not nickel and cobalt. The dual zinc and cadmium induction could be explained by the fact that both metal ions have many similar (bio)chemical characteristics. Other examples of transposase transcriptional expression induced by environmental stimuli include Tn*4652* and IS*1246* in *P. putida* KT2440 by zinc, cadmium and nickel but not cobalt (Haritha et al., 2009), and IS*Ftu1* and IS*Ftu2* by spermine in *Francisella tularensis* (Carlson et al., 2009). Elevated transpositional activity of several IS elements was also observed at high temperature in *Burkholderia multivorans* (Ohtsubo et al., 2005). However, the induced transpositional activity in the examples above did not mediate enhanced tolerance to the inducer. Although, the IS*Rme5* transposase promoter internal to the element is inducible by zinc and cadmium, the genomic region upstream IS integration has a strong regulatory effect on the expression of the IS element. The upstream genomic region resulted in reduced promoter activity of all IS*Rme5* copies, especially for IS*Rme5*b, and only the IS*Rme5*c copy was found to be metal-inducible. In addition, the transposase of IS*Rme5* (IS*481* family member) has no stop codon within the element. Consequently, the IS*Rme5*b transposase has a prolonged carboxy-terminal end compared with the other copies, generated by its insertion site, which might influence its enzymatic stability and hence, could contribute to the possible diminished transposition activity of this copy compared to the other IS*Rme5* elements. Therefore, the evolutionary success of IS*Rme*5, as probably for all IS elements, depends not only on the element itself, but also on its genomic integration site. Taking into account that many transposases preferentially act in *cis* (Mahillon and Chandler, 1998), which reduces the probability that transposase expression from a particular element would activate transposition of related copies elsewhere in the genome, the higher basal expression level and zinc-inducibility of the IS*Rme5*c transpose indicate that most IS*Rme5*-mediated CnrYX inactivation events are probably the result of IS*Rme5*c transposition. Repression or enhancement of promoter activity by the upstream flanking sequence is also observed for other MGEs (Lavie et al., 2004).

Bacteria respond to environmental changes by adapting the expression of key genes, however, such reprogramming requires time and energy. Interestingly, all tested zinc-resistant derivatives showed a considerably shorter lag phase in liquid MM284 without zinc compared to wild type, rendering the evolved population better suited to quickly respond to other environmental challenges. For instance, in conditions where the lag phase is long, adaptations reducing the lag phase are highly beneficial as the evolved population can start their exponential growth earlier and consume most of the environmental nutrients before the wild type exits the lag phase (Oxman et al., 2008). Furthermore, adaptation to zinc by IS reshuffling can have an additional impact on the adaptation to subsequent stress challenges. Insertion of an IS element in *cnrYX* in the same transcriptional orientation as the *cnr* operon will probably result in read-through transposase expression as whole-genome expression indeed verified that expression of *cnrYX* is induced in CnrH-dependent zinc-resistant AE126 derivatives. Thus, redistribution of IS elements yielding IS-dependent zinc-resistant derivatives, hence harboring an additional copy of the IS element in a constitutively transcribed region, might result in an increased pool of the transposase in the cell and subsequent enhance the adaptation potential to new environmental challenges, such as was shown here for IS*Rme5* and SacB-mediated sucrose hypersensitivity. The higher survival frequency of the IS-dependent zinc-resistant derivative compared to wild type can be explained by (i) an increased pool of IS*Rme5* transposase resulting in IS*Rme5*-mediated *sacBR* disruption, (ii) the possible insertion and subsequent excision of IS*Rme5*, resulting in various possible frameshift deletions (Ooka et al., 2009) or duplications (Mahillon and Chandler, 1998) inactivating *sacBR*, however, the presence of these mutations was not investigated, and (iii) the uncharacterized genetic background of the IS-dependent zinc-resistant derivative, which likely, consistent with other derivatives from the second population, harbors more mutations.

To summarize, it was shown that zinc induced the expression of IS elements and that these elements mediate the adaptation of *C*. *metallidurans* to zinc stress by modulating its transcriptional network or by providing outward-directed mobile promoters.

## Author contributions

Conceived and designed the study: MM, AA, and RVH. Performed the experiments: JV. Analyzed the data: all authors. Wrote the paper: JV, AA, and RVH.

## Funding

This work was supported through a Ph.D. grant for JV by SCK•CEN.

### Conflict of interest statement

The authors declare that the research was conducted in the absence of any commercial or financial relationships that could be construed as a potential conflict of interest.

## Supplementary material

Supplementary Figure 1 **Schematic representation of the *cnr* operon of megaplasmid pMOL28 from *C. metallidurans* extracted from the MaGe platform (https://www.genoscope.cns.fr/agc/microscope/mage/) with *cnrYX* encoding two membrane-bound anti-sigma factors, *cnrH* a sigma factor, *cnrCBA* a RND efflux pump, and *cnrT* a cation diffusion facilitator**. Black line represent the amplicon generated with the primer pair Cnr_Fw and Cnr_Rv used for screening the *cnrYXH* structural locus.

Supplementary Figure 2 **Promoter activity of the *cnrC* promoter of AE126 Δ*cnrH::tet*^R1^ and AE126 Δ*cnrH::tet*^R2^**. Data are represented as fold change of the report signal normalized to cell density (RLU/OD_600_) obtained for AE104 pGLR1-P_*IS*__*Rme*5*cnrC*_-*gfpluxCDABE* (953 bp fragment harboring also a 3′ fragment of IS*Rme5* without its transposase promoter) and AE104 pGLR1-P_*IS*__1086*cnrC*_-*gfpluxCDABE* (853 bp fragment harboring also a 3′ fragment of IS*1086* without its transposase promoter) compared with the control AE104 pGLR1-P_*cnrC*_-*gfpluxCDABE*.

Supplementary Table 1 **Primers used in this study**.

Supplementary Table 2 **Whole-genome microarray analysis of gene expression in two zinc-resistant AE126 derivatives (AE126^R2^ and AE126^R3^)**. Data are represented as fold change relative to the parental AE126. Only open reading frames (ORFs) that were significantly expressed (>2-fold with an adjusted *p* < 0.05) are shown. **(A)** ORFs differentially expressed in both derivatives, **(B)** ORFs differentially expressed in AE126^R2^ and **(C)** ORFs differentially expressed in AE126^R3^ with ND not determined. The full description of the microarray data have been deposited at the Gene Expression Omnibus website (http://www.ncbi.nlm.nih.gov/geo/) under accession number GSE74091.

Supplementary Table 3 **Scan of the *C. metallidurans* CH34 genome sequence (+ for forward and – for reverse strand) with the position-specific scoring matrix obtained from the multiple sequence alignment of the ISRme5 target sites in 87 zinc-resistant AE126 derivatives using RSAT**. The weight score Ws = log (P(S|M)/P(S|B)) is the probability for the sequence segment S to occur according to the motif model M, whereas P (S|B) is the probability for the same sequence S to occur under the background model B. The weight score is thus the log-ratio of the likelihood of S in these two respective models. Top 100 targets are shown.

Supplementary Table 4 **Transcriptional expression of the *cnrCBAT* cluster, determined by whole-microarray analysis, in two zinc-resistant AE126 derivatives (AE126^R2^ and AE126^R3^) and in a zinc-resistant AE126 Δ*cnrH::tet* derivative (AE126Δ*cnrH::tet*^R1^)**. Data are represented as fold change relative to the parental AE126 and AE126 Δ*cnrH::tet*, respectively. The full description of the microarray data have been deposited at the Gene Expression Omnibus website (http://www.ncbi.nlm.nih.gov/geo/) under accession number GSE74091.

## References

[B1] Baranyi J. Roberts T. A. (1994). A dynamic approach to predicting bacterial growth in food. Int. J. Food Microbiol. 23, 277–294. 10.1016/0168-1605(94)90157-0 7873331

[B2] Benedetti I. M. de Lorenzo V. Silva-Rocha R. (2012). Quantitative, non-disruptive monitoring of transcription in single cells with a broad-host range GFP-luxCDABE dual reporter system. PLoS ONE 7:e52000. 10.1371/journal.pone.0052000 23284849 PMC3532404

[B3] Brim H. Heyndrickx M. de Vos P. Wilmotte A. Springael D. Schlegel H. G.. (1999). Amplified rDNA restriction analysis and further genotypic characterisation of metal-resistant soil bacteria and related facultative hydrogenotrophs. Syst. Appl. Microbiol. 22, 258–268. 10.1016/S0723-2020(99)80073-3 10390877

[B4] Cairns J. Overbaugh J. Miller S. (1988). The origin of mutants. Nature 335, 142–145. 10.1038/335142a0 3045565

[B5] Carlson P. E. Jr. Horzempa J. O'Dee D. M. Robinson C. M. Neophytou P. Labrinidis A.. (2009). Global transcriptional response to spermine, a component of the intramacrophage environment, reveals regulation of *Francisella* gene expression through insertion sequence elements. J. Bacteriol. 191, 6855–6864. 10.1128/JB.00995-09 19749055 PMC2772466

[B6] Chang A. C. Cohen S. N. (1978). Construction and characterization of amplifiable multicopy DNA cloning vehicles derived from the P15A cryptic miniplasmid. J. Bacteriol. 134, 1141–1156. 149110 10.1128/jb.134.3.1141-1156.1978 PMC222365

[B7] Christie-Oleza J. A. Lanfranconi M. P. Nogales B. Lalucat J. Bosch R. (2009). Conjugative interaction induces transposition of IS*Pst9* in *Pseudomonas stutzeri* AN10. J. Bacteriol. 191, 1239–1247. 10.1128/JB.01071-08 19060139 PMC2631995

[B8] Ciampi M. S. Schmid M. B. Roth J. R. (1982). Transposon Tn*10* provides a promoter for transcription of adjacent sequences. Proc. Natl. Acad. Sci. U.S.A. 79, 5016–5020. 10.1073/pnas.79.16.5016 6289329 PMC346817

[B9] Coenye T. Spilker T. Reik R. Vandamme P. Lipuma J. J. (2005). Use of PCR analyses to define the distribution of *Ralstonia* species recovered from patients with cystic fibrosis. J. Clin. Microbiol. 43, 3463–3466. 10.1128/JCM.43.7.3463-3466.2005 16000479 PMC1169115

[B10] Collard J. M. Provoost A. Taghavi S. Mergeay M. (1993). A new type of *Alcaligenes eutrophus* CH34 zinc resistance generated by mutations affecting regulation of the *cnr* cobalt-nickel resistance system. J. Bacteriol. 175, 779–784. 8423150 10.1128/jb.175.3.779-784.1993 PMC196217

[B11] Craig N. L. (1997). Target site selection in transposition. Annu. Rev. Biochem. 66, 437–474. 10.1146/annurev.biochem.66.1.437 9242914

[B12] Crooks G. E. Hon G. Chandonia J. M. Brenner S. E. (2004). WebLogo: a sequence logo generator. Genome Res. 14, 1188–1190. 10.1101/gr.849004 15173120 PMC419797

[B13] Dallmeier K. Neyts J. (2013). Simple and inexpensive three-step rapid amplification of cDNA 5′ ends using 5′ phosphorylated primers. Anal. Biochem. 434, 1–3. 10.1016/j.ab.2012.10.031 23123427 PMC3562438

[B14] Darwin C. (1910). The Origin of Species by Means of Natural Selection. London: London John Murray.

[B15] Del Re B. Garoia F. Mesirca P. Agostini C. Bersani F. Giorgi G. (2003). Extremely low frequency magnetic fields affect transposition activity in *Escherichia coli*. Radiat. Environ. Biophys. 42, 113–118. 10.1007/s00411-003-0192-9 12768290

[B16] Diels L. Mergeay M. (1990). DNA probe-mediated detection of resistant bacteria from soils highly polluted by heavy metals. Appl. Environ. Microbiol. 56, 1485–1491. 16348196 10.1128/aem.56.5.1485-1491.1990 PMC184435

[B17] D'Inzeo T. Santangelo R. Fiori B. De Angelis G. Conte V. Giaquinto A.. (2015). Catheter-related bacteremia by *Cupriavidus metallidurans*. Diagn. Microbiol. Infect. Dis. 81, 9–12. 10.1016/j.diagmicrobio.2014.09.015 25446890

[B18] Dong Q. Sadouk A. van der Lelie D. Taghavi S. Ferhat A. Nuyten J. M.. (1992). Cloning and sequencing of IS*1086*, an *Alcaligenes eutrophus* insertion element related to IS*30* and IS*4351*. J. Bacteriol. 174, 8133–8138. 1334071 10.1128/jb.174.24.8133-8138.1992 PMC207552

[B19] Drevinek P. Baldwin A. Lindenburg L. Joshi L. T. Marchbank A. Vosahlikova S.. (2010). Oxidative stress of *Burkholderia cenocepacia* induces insertion sequence-mediated genomic rearrangements that interfere with macrorestriction-based genotyping. J. Clin. Microbiol. 48, 34–40. 10.1128/JCM.01433-09 19889907 PMC2812269

[B20] Eichenbaum Z. Livneh Z. (1998). UV light induces IS*10* transposition in *Escherichia coli*. Genetics 149, 1173–1181. 9649512 10.1093/genetics/149.3.1173 PMC1460249

[B21] Foster P. L. (2007). Stress-induced mutagenesis in bacteria. Crit. Rev. Biochem. Mol. Biol. 42, 373–397. 10.1080/10409230701648494 17917873 PMC2747772

[B22] Gay P. Le Coq D. Steinmetz M. Berkelman T. Kado C. I. (1985). Positive selection procedure for entrapment of insertion sequence elements in gram-negative bacteria. J. Bacteriol. 164, 918–921. 2997137 10.1128/jb.164.2.918-921.1985 PMC214340

[B23] González J. Karasov T. L. Messer P. W. Petrov D. A. (2010). Genome-wide patterns of adaptation to temperate environments associated with transposable elements in *Drosophila*. PLoS Genet. 6:e1000905. 10.1371/journal.pgen.1000905 20386746 PMC2851572

[B24] Goris J. De Vos P. Coenye T. Hoste B. Janssens D. Brim. (2001). Classification of metal-resistant bacteria from industrial biotopes as *Ralstonia campinensis* sp nov., *Ralstonia metallidurans* sp nov and *Ralstonia basilensis* Steinle et al. 1998 emend. Int. J. Syst. Evol. Micr. 51, 1773–1782. 10.1099/00207713-51-5-1773 11594608

[B25] Grass G. Grosse C. Nies D. H. (2000). Regulation of the *cnr* cobalt and nickel resistance determinant from *Ralstonia* sp. strain CH34. J. Bacteriol. 182, 1390–1398. 10.1128/JB.182.5.1390-1398.2000 10671463 PMC94428

[B26] Grosse C. Friedrich S. Nies D. H. (2007). Contribution of extracytoplasmic function sigma factors to transition metal homeostasis in *Cupriavidus metallidurans* strain CH34. J. Mol. Microbiol. Biotechnol. 12, 227–240. 10.1159/000099644 17587871

[B27] Hall B. G. (1990). Directed evolution of a bacterial operon. Bioessays 12, 551–558. 10.1002/bies.950121109 2085322

[B28] Hall B. M. Ma C. X. Liang P. Singh K. K. (2009). Fluctuation analysis CalculatOR: a web tool for the determination of mutation rate using Luria-Delbruck fluctuation analysis. Bioinformatics 25, 1564–1565. 10.1093/bioinformatics/btp253 19369502 PMC2687991

[B29] Haritha A. Sagar K. P. Tiwari A. Kiranmayi P. Rodrigue A. Mohan P. M.. (2009). MrdH, a novel metal resistance determinant of *Pseudomonas putida* KT2440, is flanked by metal-inducible mobile genetic elements. J. Bacteriol. 191, 5976–5987. 10.1128/JB.00465-09 19648243 PMC2747888

[B30] Janssen P. J. Van Houdt R. Moors H. Monsieurs P. Morin N. Michaux A.. (2010). The complete genome sequence of *Cupriavidus metallidurans* strain CH34, a master survivalist in harsh and anthropogenic environments. PLoS ONE 5:e10433. 10.1371/journal.pone.0010433 20463976 PMC2864759

[B31] Jaurin B. Normark S. (1983). Insertion of IS*2* creates a novel *ampC* promoter in *Escherichia coli*. Cell 32, 809–816. 10.1016/0092-8674(83)90067-3 6187472

[B32] Kasak L. Horak R. Kivisaar M. (1997). Promoter-creating mutations in *Pseudomonas putida*: a model system for the study of mutation in starving bacteria. Proc. Natl. Acad. Sci. U.S.A. 94, 3134–3139. 10.1073/pnas.94.7.3134 9096358 PMC20334

[B33] Katzen F. Becker A. Ielmini M. V. Oddo C. G. Ielpi L. (1999). New mobilizable vectors suitable for gene replacement in gram-negative bacteria and their use in mapping of the 3′ end of the *Xanthomonas campestris* pv. campestris *gum* operon. Appl Environ Microb 65, 278–282. 9872790 10.1128/aem.65.1.278-282.1999 PMC91013

[B34] Kazazian H. H. Jr. (2004). Mobile elements: drivers of genome evolution. Science 303, 1626–1632. 10.1126/science.1089670 15016989

[B35] Kovach M. E. Elzer P. H. Hill D. S. Robertson G. T. Farris M. A. Roop R. M. II. (1995). Four new derivatives of the broad-host-range cloning vector pBBR1MCS, carrying different antibiotic-resistance cassettes. Gene 166, 175–176. 10.1016/0378-1119(95)00584-1 8529885

[B36] Lampe D. J. Grant T. E. Robertson H. M. (1998). Factors affecting transposition of the Himar1 mariner transposon *in vitro*. Genetics 149, 179–187. 9584095 10.1093/genetics/149.1.179 PMC1460121

[B37] Langevin S. Vincelette J. Bekal S. Gaudreau C. (2011). First case of invasive human infection caused by *Cupriavidus metallidurans*. J. Clin. Microbiol. 49, 744–745. 10.1128/JCM.01947-10 21106795 PMC3043494

[B38] Lartigue M. F. Poirel L. Aubert D. Nordmann P. (2006). *In vitro* analysis of IS*Ecp1B*-mediated mobilization of naturally occurring beta-lactamase gene bla_CTX−M_ of *Kluyvera ascorbata*. Antimicrob. Agents Chemother. 50, 1282–1286. 10.1128/AAC.50.4.1282-1286.2006 16569841 PMC1426957

[B39] Lavie L. Maldener E. Brouha B. Meese E. U. Mayer J. (2004). The human L1 promoter: variable transcription initiation sites and a major impact of upstream flanking sequence on promoter activity. Genome Res. 14, 2253–2260. 10.1101/gr.2745804 15520289 PMC525683

[B40] Lederberg J. (1989). Replica plating and indirect selection of bacterial mutants: isolation of preadaptive mutants in bacteria by sib selection. Genetics 121, 395–399. 2653959 10.1093/genetics/121.3.395 PMC1203627

[B41] Liao G. C. Rehm E. J. Rubin G. M. (2000). Insertion site preferences of the P transposable element in *Drosophila melanogaster*. Proc. Natl. Acad. Sci. U.S.A. 97, 3347–3351. 10.1073/pnas.97.7.3347 10716700 PMC16242

[B42] Louarn J. M. Bouche J. P. Legendre F. Louarn J. Patte J. (1985). Characterization and properties of very large inversions of the *Escherichia coli* chromosome along the origin-to-terminus axis. Mol. Gen. Genet. 201, 467–476. 10.1007/BF00331341 3911026

[B43] Luria S. E. Delbrück M. (1943). Mutations of bacteria from virus sensitivity to virus resistance. Genetics 28, 491–511. 17247100 10.1093/genetics/28.6.491 PMC1209226

[B44] MacLean R. C. Torres-Barceló C. Moxon R. (2013). Evaluating evolutionary models of stress-induced mutagenesis in bacteria. Nat. Rev. Genet. 14, 221–227. 10.1038/nrg3415 23400102

[B45] Ma-e-Talat (2000). Genetic Mechanism of Heavy Metal Resistance of Pseudomonas aeruginosa Cmg103. Karachi: University of Karachi.

[B46] Mahillon J. Chandler M. (1998). Insertion sequences. Microbiol. Mol. Biol. Rev. 62, 725–774. 9729608 10.1128/mmbr.62.3.725-774.1998 PMC98933

[B47] Mahillon J. Léonard C. Chandler M. (1999). IS elements as constituents of bacterial genomes. Res. Microbiol. 150, 675–687. 10.1016/S0923-2508(99)00124-2 10673006

[B48] Maillard A. P. Künnemann S. Grosse C. Volbeda A. Schleuder G. Petit-Härtlein I.. (2015). Response of CnrX from *Cupriavidus metallidurans* CH34 to nickel binding. Metallomics 7, 622–631. 10.1039/C4MT00293H 25628016

[B49] Maruyama I. N. Rakow T. L. Maruyama H. I. (1995). Crace - a simple method for identification of the 5′-end of messenger-RNAs. Nucleic Acids Res. 23, 3796–3797. 10.1093/nar/23.18.3796 7479016 PMC307285

[B50] McClintock B. (1984). The significance of responses of the genome to challenge. Science 226, 792–801. 10.1126/science.15739260 15739260

[B51] Mergeay M. Monchy S. Vallaeys T. Auquier V. Benotmane A. Bertin P.. (2003). *Ralstonia metallidurans*, a bacterium specifically adapted to toxic metals: towards a catalogue of metal-responsive genes. FEMS Microbiol. Rev. 27, 385–410. 10.1016/S0168-6445(03)00045-7 12829276

[B52] Mergeay M. Nies D. Schlegel H. G. Gerits J. Charles P. Vangijsegem F. (1985). *Alcaligenes eutrophus* CH34 is a facultative chemolithotroph with plasmid-bound resistance to heavy-metals. J. Bacteriol. 162, 328–334. 3884593 10.1128/jb.162.1.328-334.1985 PMC218993

[B53] Mijnendonckx K. Provoost A. Monsieurs P. Leys N. Mergeay M. Mahillon J.. (2011). Insertion sequence elements in *Cupriavidus metallidurans* CH34: distribution and role in adaptation. Plasmid 65, 193–203. 10.1016/j.plasmid.2010.12.006 21185859

[B54] Mijnendonckx K. Provoost A. Ott C. M. Venkateswaran K. Mahillon J. Leys N.. (2013). Characterization of the survival ability of *Cupriavidus metallidurans* and *Ralstonia pickettii* from space-related environments. Microb. Ecol. 65, 347–360. 10.1007/s00248-012-0139-2 23212653

[B55] Monchy S. Benotmane M. A. Janssen P. Vallaeys T. Taghavi S. van der Lelie D.. (2007). Plasmids pMOL28 and pMOL30 of *Cupriavidus metallidurans* are specialized in the maximal viable response to heavy metals. J. Bacteriol. 189, 7417–7425. 10.1128/JB.00375-07 17675385 PMC2168447

[B56] Monsieurs P. Moors H. Van Houdt R. Janssen P. J. Janssen A. Coninx I.. (2011). Heavy metal resistance in *Cupriavidus metallidurans* CH34 is governed by an intricate transcriptional network. Biometals 24, 1133–1151. 10.1007/s10534-011-9473-y 21706166

[B57] Nies D. H. (1999). Microbial heavy-metal resistance. Appl. Microbiol. Biotechnol. 51, 730–750. 10.1007/s002530051457 10422221

[B58] Nies D. H. (2003). Efflux-mediated heavy metal resistance in prokaryotes. FEMS Microbiol. Rev. 27, 313–339. 10.1016/S0168-6445(03)00048-2 12829273

[B59] Nies D. H. Silver S. (1995). Ion efflux systems involved in bacterial metal resistances. J. Ind. Microbiol. 14, 186–199. 10.1007/BF01569902 7766211

[B60] Ohtsubo Y. Genka H. Komatsu H. Nagata Y. Tsuda M. (2005). High-temperature-induced transposition of insertion elements in *Burkholderia multivorans* ATCC 17616. Appl. Environ. Microbiol. 71, 1822–1828. 10.1128/AEM.71.4.1822-1828.2005 15812007 PMC1082539

[B61] Ooka T. Ogura Y. Asadulghani M. Ohnishi M. Nakayama K. Terajima J.. (2009). Inference of the impact of insertion sequence (IS) elements on bacterial genome diversification through analysis of small-size structural polymorphisms in *Escherichia coli* O157 genomes. Genome Res. 19, 1809–1816. 10.1101/gr.089615.108 19564451 PMC2765283

[B62] Oxman E. Alon U. Dekel E. (2008). Defined order of evolutionary adaptations: experimental evidence. Evolution 62, 1547–1554. 10.1111/j.1558-5646.2008.00397.x 18410537

[B63] Parkhill J. Sebaihia M. Preston A. Murphy L. D. Thomson N. Harris D. E.. (2003). Comparative analysis of the genome sequences of *Bordetella pertussis, Bordetella parapertussis* and *Bordetella bronchiseptica*. Nat. Genet. 35, 32–40. 10.1038/ng1227 12910271

[B64] Pasternak C. Ton-Hoang B. Coste G. Bailone A. Chandler M. Sommer S. (2010). Irradiation-induced *Deinococcus radiodurans* genome fragmentation triggers transposition of a single resident insertion sequence. PLoS Genet. 6:e1000799. 10.1371/journal.pgen.1000799 20090938 PMC2806898

[B65] Prentki P. Teter B. Chandler M. Galas D. J. (1986). Functional promoters created by the insertion of transposable element IS*1*. J. Mol. Biol. 191, 383–393. 10.1016/0022-2836(86)90134-8 3029382

[B66] Ram Y. Hadany L. (2014). Stress-induced mutagenesis and complex adaptation. Proc. R. Soc. B 281:20141025. 10.1098/rspb.2014.1025 25143032 PMC4150318

[B67] Redenbach M. Kieser H. M. Denapaite D. Eichner A. Cullum J. Kinashi H.. (1996). A set of ordered cosmids and a detailed genetic and physical map for the 8 Mb *Streptomyces coelicolor* A3(2) chromosome. Mol. Microbiol. 21, 77–96. 10.1046/j.1365-2958.1996.6191336.x 8843436

[B68] Riesenfeld C. Everett M. Piddock L. J. Hall B. G. (1997). Adaptive mutations produce resistance to ciprofloxacin. Antimicrob. Agents Chemother. 41, 2059–2060. 9303418 10.1128/aac.41.9.2059 PMC164069

[B69] Rosenberg S. M. Hastings P. J. (2004). Adaptive point mutation and adaptive amplification pathways in the *Escherichia coli* Lac system: stress responses producing genetic change. J. Bacteriol. 186, 4838–4843. 10.1128/JB.186.15.4838-4843.2004 15262914 PMC451650

[B70] Roth J. R. (2011). The joys and terrors of fast adaptation: new findings elucidate antibiotic resistance and natural selection. Mol. Microbiol. 79, 279–282. 10.1111/j.1365-2958.2010.07459.x 21219449 PMC3064428

[B71] Saier M. H. Jr. Zhang Z. (2014). Transposon-mediated directed mutation controlled by DNA binding proteins in *Escherichia coli*. Front. Microbiol. 5:390. 10.3389/fmicb.2014.00390 25136335 PMC4117983

[B72] Schneider D. Duperchy E. Coursange E. Lenski R. E. Blot M. (2000a). Long-term experimental evolution in *Escherichia coli*. IX. Characterization of insertion sequence-mediated mutations and rearrangements. Genetics 156, 477–488. 11014799 10.1093/genetics/156.2.477 PMC1461276

[B73] Schneider D. Faure D. Noirclerc-Savoye M. Barrière A. C. Coursange E. Blot M. (2000b). A broad-host-range plasmid for isolating mobile genetic elements in gram-negative bacteria. Plasmid 44, 201–207. 10.1006/plas.2000.1483 10964631

[B74] Schroeder A. Mueller O. Stocker S. Salowsky R. Leiber M. Gassmann M.. (2006). The RIN: an RNA integrity number for assigning integrity values to RNA measurements. BMC Mol. Biol. 7:3. 10.1186/1471-2199-7-3 16448564 PMC1413964

[B75] Shapiro J. A. (1984). Observations on the formation of clones containing *araB*-*lacZ* cistron fusions. Mol. Gen. Genet. 194, 79–90. 10.1007/BF00383501 6233472

[B76] Siguier P. Filée J. Chandler M. (2006). Insertion sequences in prokaryotic genomes. Curr. Opin. Microbiol. 9, 526–531. 10.1016/j.mib.2006.08.005 16935554

[B77] Siguier P. Gourbeyre E. Chandler M. (2014). Bacterial insertion sequences: their genomic impact and diversity. FEMS Microbiol. Rev. 38, 865–891. 10.1111/1574-6976.12067 24499397 PMC7190074

[B78] Stanley D. Fraser S. Stanley G. A. Chambers P. J. (2010). Retrotransposon expression in ethanol-stressed *Saccharomyces cerevisiae*. Appl. Microbiol. Biotechnol. 87, 1447–1454. 10.1007/s00253-010-2562-y 20393705

[B79] Studer A. Zhao Q. Ross-Ibarra J. Doebley J. (2011). Identification of a functional transposon insertion in the maize domestication gene *tb1*. Nat. Genet. 43, 1160–1163. 10.1038/ng.942 21946354 PMC3686474

[B80] Takahashi K. Sekine Y. Chibazakura T. Yoshikawa H. (2007). Development of an intermolecular transposition assay system in *Bacillus subtilis* 168 using IS*4Bsu1* from *Bacillus subtilis* (natto). Microbiology 153, 2553–2559. 10.1099/mic.0.2007/007104-0 17660419

[B81] Tauch A. Zheng Z. Pühler A. Kalinowski J. (1998). Corynebacterium striatum chloramphenicol resistance transposon Tn*5564*: genetic organization and transposition in *Corynebacterium glutamicum*. Plasmid 40, 126–139. 10.1006/plas.1998.1362 9735314

[B82] Tibazarwa C. Wuertz S. Mergeay M. Wyns L. van Der Lelie D. (2000). Regulation of the *cnr* cobalt and nickel resistance determinant of *Ralstonia eutropha* (*Alcaligenes eutrophus*) CH34. J. Bacteriol. 182, 1399–1409. 10.1128/JB.182.5.1399-1409.2000 10671464 PMC94429

[B83] Trepreau J. Girard E. Maillard A. P. de Rosny E. Petit-Haertlein I. Kahn R.. (2011). Structural basis for metal sensing by CnrX. J. Mol. Biol. 408, 766–779. 10.1016/j.jmb.2011.03.014 21414325

[B84] Trepreau J. Grosse C. Mouesca J. M. Sarret G. Girard E. Petit-Haertlein I.. (2014). Metal sensing and signal transduction by CnrX from *Cupriavidus metallidurans* CH34: role of the only methionine assessed by a functional, spectroscopic, and theoretical study. Metallomics 6, 263–273. 10.1039/C3MT00248A 24154823

[B85] Turatsinze J. V. Thomas-Chollier M. Defrance M. van Helden J. (2008). Using RSAT to scan genome sequences for transcription factor binding sites and cis-regulatory modules. Nat. Protoc. 3, 1578–1588. 10.1038/nprot.2008.97 18802439

[B86] Van Houdt R. Monchy S. Leys N. Mergeay M. (2009). New mobile genetic elements in *Cupriavidus metallidurans* CH34, their possible roles and occurrence in other bacteria. Antonie Van Leeuwenhoek 96, 205–226. 10.1007/s10482-009-9345-4 19390985

[B87] Van Houdt R. Monsieurs P. Mijnendonckx K. Provoost A. Janssen A. Mergeay M.. (2012). Variation in genomic islands contribute to genome plasticity in *Cupriavidus metallidurans*. BMC Genomics 13:111. 10.1186/1471-2164-13-111 22443515 PMC3384475

[B88] von Rozycki T. Nies D. H. (2009). *Cupriavidus metallidurans*: evolution of a metal-resistant bacterium. Antonie Van Leeuwenhoek 96, 115–139. 10.1007/s10482-008-9284-5 18830684

[B89] Wang X. Wood T. K. (2011). IS*5* inserts upstream of the master motility operon *flhDC* in a quasi-Lamarckian way. ISME J. 5, 1517–1525. 10.1038/ismej.2011.27 21390082 PMC3160685

[B90] Wright B. E. (2004). Stress-directed adaptive mutations and evolution. Mol. Microbiol. 52, 643–650. 10.1111/j.1365-2958.2004.04012.x 15101972

[B91] Zhang Z. Saier M. H. Jr. (2009). A mechanism of transposon-mediated directed mutation. Mol. Microbiol. 74, 29–43. 10.1111/j.1365-2958.2009.06831.x 19682247 PMC2973706

[B92] Zhang Z. Saier M. H. Jr. (2011). Transposon-mediated adaptive and directed mutations and their potential evolutionary benefits. J. Mol. Microbiol. Biotechnol. 21, 59–70. 10.1159/000333108 22248543 PMC3697268

