# Supplementary figures and images for: Zinc-Induced Transposition of Insertion Sequence Elements Contributes to Increased Adaptability of Cupriavidus metallidurans
Source: Front Microbiol. 2016 Mar 23;7:359. doi: 10.3389/fmicb.2016.00359 (PMC4803752; doi:10.3389/fmicb.2016.00359)

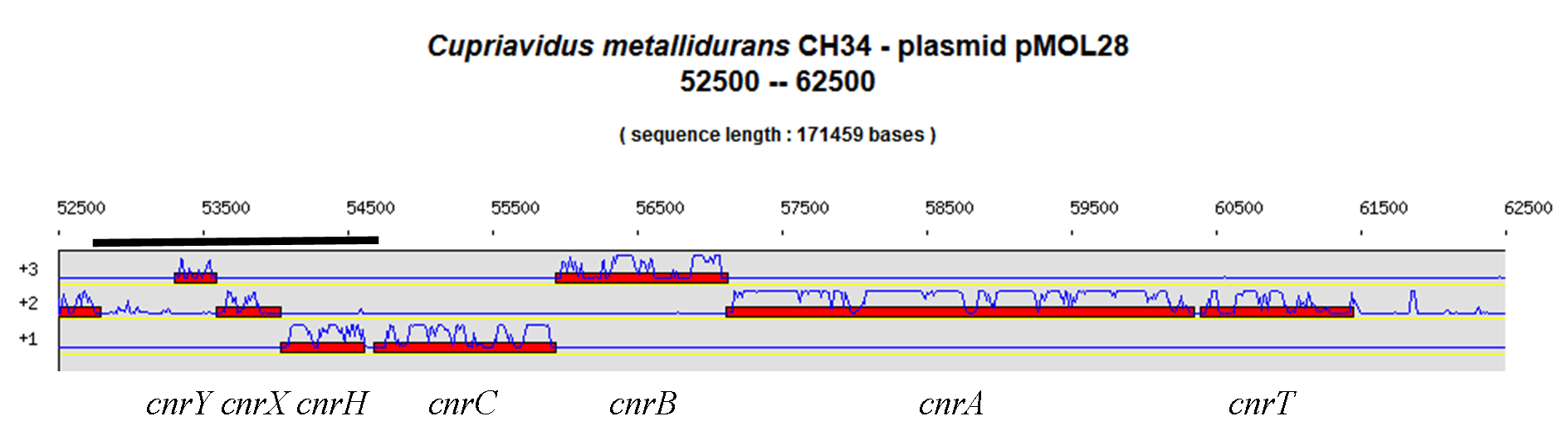

Supplement: Supplementary Figure 1 — Schematic representation of the cnr operon of megaplasmid pMOL28 from C. metallidurans extracted from the MaGe platform (https://www.genoscope.cns.fr/agc/microscope/mage/) with cnrYX encoding two membrane-bound anti-sigma factors, cnrH a sigma factor, cnrCBA a RND efflux pump, and cnrT a cation diffusion facilitator. Black line represent the amplicon generated with the primer pair Cnr_Fw and Cnr_Rv used for screening the cnrYXH structural locus. [file Image1.TIF]

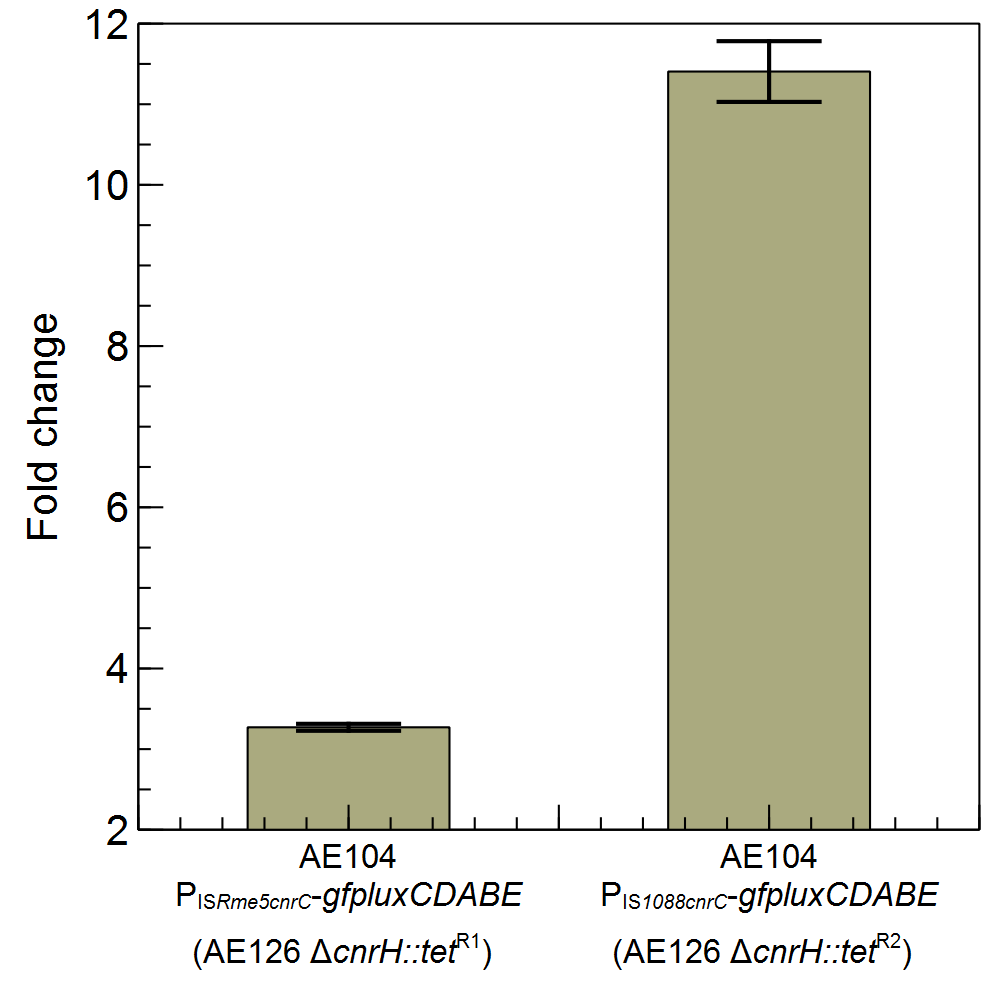

Supplement: Supplementary Figure 2 — Promoter activity of the cnrC promoter of AE126 ΔcnrH::tetR1 and AE126 ΔcnrH::tetR2. Data are represented as fold change of the report signal normalized to cell density (RLU/OD600) obtained for AE104 pGLR1-PISRme5cnrC-gfpluxCDABE (953 bp fragment harboring also a 3′ fragment of ISRme5 without its transposase promoter) and AE104 pGLR1-PIS1086cnrC-gfpluxCDABE (853 bp fragment harboring also a 3′ fragment of IS1086 without its transposase promoter) compared with the control AE104 pGLR1-PcnrC-gfpluxCDABE. [file Image2.TIFF]
