# Supplementary material for: Zinc-Induced Transposition of Insertion Sequence Elements Contributes to Increased Adaptability of Cupriavidus metallidurans
Source: Front Microbiol. 2016 Mar 23;7:359. doi: 10.3389/fmicb.2016.00359 (PMC4803752; doi:10.3389/fmicb.2016.00359)
Supplement: Supplementary Table 1 — Primers used in this study. [file Table1.DOCX]

| Supplementary Table 1. Primers used in this study | | |
| --- | --- | --- |
| Primer pair | 5’-3’-Sequence | |
| Cnr_Fw | GTAAATCGCAATGGGCTTATCA | |
| Cnr_Rv | ACGAGGAGATCACCTGTTTCAT | |
| SacR_Rv | TCAGCAACACCTTCTTCACG | |
| IS*Rme5*_Fw | CATCTGAGGCTGCACAAGAA | |
| pIS*Rme5*_Fw | ACGTGAATTCTGTCGTGTCCCTGATGATTGG | |
| pIS*Rme5*a_Fw | ACGTGAATTCCGCATCCAGCCACAAAACAT | |
| pIS*Rme5*b_Fw | ACGTGAATTCAACCAGTGTTCGTTCAGGCA | |
| pIS*Rme5*c_Fw | CTAGGAATTCTGGTATTCGATGTTGGCATG | |
| pIS*Rme5*d_Fw | GATCGAATTCTTAGGGGCATGGTCCGATTG | |
| pIS*Rme5*_Rv | CAACTCTAGACTCAGATGCATCGTTTCAGC | |
| CnrIns_Fw | CGTAGAGCTCGTAAATCGCAATGGGCTTATCA | |
| CnrIns_Rv | GCTAACTAGTACGAGGAGATCACCTGTTTCAT | |
| CnrT_Rv | GAGTACTAGTTACGCGCATCGACTACTTCC | |
| Cnr_FwpBBR1MCS2 | CACTATAGGGCGAATTGGTGAGAATGATGGCAATCCGTTTC | |
| Cnr_RvpBBR1MCS2 | AAGGGAACAAAAGCTGTACGCGCATCGACTACTTCCGAGT | |
| pBBR1MCS2_Fw | CAGCTTTTGTTCCCTTTAGTGAG | |
| pBBR1MCS2_Rv | AATTCGCCCTATAGTGAGTCGTAT | |
| pIS*Rme5_*Fw | CTAGGAATTCTGGTATTCGATGTTGGCATG | |
| pIS*Rme5_*Rv | CAACTCTAGACTCAGATGCATCGTTTCAGC | |
| pIS*1088_*Fw | GTTCGAATTCTCGACTTCTATCGCGTCGAG | |
| pIS*1088_*Rv | CTAGTCTAGAGCGTTCGGTCTCACTCAACT | |
| pIS*1087B_*Fw | CTAGGAATTCGGCAACTTTGAGCGACATCC | |
| pIS*1087B_*Rv | CTAGTCTAGACCACGATTTGCCTAGGCTTG | |
| CnrC_R1 (5’-Phosphate) | AAGCGATTGCTGCAACAGG | |
| CnrC_R2 | GAGGTCGACAAAGGAACGAG | |
| CnrC_F1 | TATGGCTGCTGCCCGTTG | |
| pCnrC_Fw | CACCGACAAACAACAGGTGAGAATGATGGCAATCCGTTTC | |
| pCnrC_Rv | TCTAGAGGATCCCCGGCAAGAGGTCAGCGGTGGAGGC | |
| IS*Rme5*_Internal | CACCGACAAACAACAGCTGAGGCTGCACAAGAACGAC | |
| IS*1086*_Internal | CACCGACAAACAACAGCTCGCTCCTGATGGCGTATT | |
| pGLR1_Fw | CCGGGGATCCTCTAGAGTCGA | |
| pGLR1_Rv | CTGTTGTTTGTCGGTGAACGC | |
| Restriction sites are underlined; homologous ends are double underlined | |  |
