# Supplementary material for: Zinc-Induced Transposition of Insertion Sequence Elements Contributes to Increased Adaptability of Cupriavidus metallidurans
Source: Front Microbiol. 2016 Mar 23;7:359. doi: 10.3389/fmicb.2016.00359 (PMC4803752; doi:10.3389/fmicb.2016.00359)
Supplement: Supplementary Table 2 — Whole-genome microarray analysis of gene expression in two zinc-resistant AE126 derivatives (AE126R2 and AE126R3). Data are represented as fold change relative to the parental AE126. Only open reading frames (ORFs) that were significantly expressed (>2-fold with an adjusted p < 0.05) are shown. (A) ORFs differentially expressed in both derivatives, (B) ORFs differentially expressed in AE126R2 and (C) ORFs differentially expressed in AE126R3 with ND not determined. The full description of the microarray data have been deposited at the Gene Expression Omnibus website (http://www.ncbi.nlm.nih.gov/geo/) under accession number GSE74091. [file Table2.DOCX]

| Supplementary Table 2. Whole-genome microarray analysis of gene expression in two zinc-resistant AE126 derivatives (AE126^R2^ and AE126^R3^). Data are represented as fold change relative to the parental AE126. Only open reading frames (ORFs) that were significantly expressed (> 2-fold with an adjusted p-value < 0.05) are shown. (A) ORFs differentially expressed in both derivatives, (B) ORFs differentially expressed in AE126^R2^ and (C) ORFs differentially expressed in AE126^R3^ with ND not determined. The full description of the microarray data have been deposited at the Gene Expression Omnibus website (http://www.ncbi.nlm.nih.gov/geo/) under accession number GSE74091. | | | | | | | |
| --- | --- | --- | --- | --- | --- | --- | --- |
| **A)** |  |  |  | |  | | |
| **Rmet** | **Gene** | **Protein Function** | **R^2^** | | **R^3^** | | |
| Rmet_6206 | *cnrX* | Anti-sigma factor involved in Co(II) and Ni(II) response | 15.45 | | 56.76 | | |
| Rmet_6209 | *cnrB* | Membrane fusion protein. three components cation proton antiporter efflux system. involved in Co(II) and Ni(II) resistance | 11.77 | | 20.48 | | |
| Rmet_6205 | *cnrY* | Anti-sigma factor involved in Co(II) and Ni(II) response | 11.29 | | 33.00 | | |
| Rmet_6210 | *cnrA* | Inner membrane efflux pump, three components cation proton antiporter efflux system. involved in Co(II) and Ni(II) resistance | 9.68 | | 21.69 | | |
| Rmet_6211 | *cnrT* | Cation Diffusion Facilitator, involved in Co(II) and Ni(II) resistance | 8.06 | | 18.78 | | |
| Rmet_6208 | *cnrC* | Outer membrane protein, three components cation proton antiporter efflux system. involved in Co(II) and Ni(II) resistance | 6.29 | | 10.19 | | |
| Rmet_6269 | Rmet_6269 | Conserved hypothetical protein | 3.09 | | 3.14 | | |
| Rmet_6207 | *cnrH* | Sigma factor, involved in Co(II) and Ni(II) response | 3.05 | | 5.92 | | |
| Rmet_3376 | *kefC* | Potassium:proton antiporter | 2.99 | | 2.70 | | |
| Rmet_3786 | Rmet_3786 | Putative outer membrane porin | 2.79 | | 2.02 | | |
| Rmet_1983 | Rmet_1983 | Hypothetical protein | 2.47 | | 2.84 | | |
| Rmet_1364 | *metC* | Cystathionine beta-lyase, PLP-dependent | 2.39 | | 2.18 | | |
| Rmet_0194 | *ribA* | GTP cyclohydrolase II | 2.25 | | 2.36 | | |
| Rmet_5868 | *leuC* | 3-isopropylmalate dehydratase large subunit | 2.22 | | 2.28 | | |
| Rmet_5401 | Rmet_5401 | Putative transmembrane transcriptional regulator (anti-sigma factor) | 2.22 | | 2.48 | | |
| Rmet_6185 | *merE* | MerE from Tn4378, membrane protein involved in Hg(II) resistance | 2.19 | | 2.42 | | |
| Rmet_1767 | Rmet_1767 | Transcriptional regulator, LysR family | 2.07 | | 2.47 | | |
| Rmet_1363 | Rmet_1363 | Conserved hypothetical protein | 2.01 | | 2.01 | | |
| Rmet_4596 | *czcC2* | Heavy metal cation tricomponent efflux outer membrane porin | 0.28 | | 0.34 | | |
| **B)** |  |  |  | |  | |  |
| **Rmet** | **Gene** | **Protein Function** | **R^2^** | | **R^3^** | |  |
| Rmet_2994 | *ptxB* | Phosphite transport system-binding protein ptxB precursor | 16.92 | | 0.40 | |  |
| Rmet_2992 | *ptxD* | Phosphonate dehydrogenase (NAD-dependent phosphite dehydrogenase) | 11.90 | | 0.27 | |  |
| Rmet_2993 | *ptxC* | Phosphite transport system permease protein PtxC | 10.67 | | 0.41 | |  |
| Rmet_2995 | *ptxA* | Phosphonate/organophosphate ester transporter subunit ; ATP-binding component of ABC superfamily | 10.37 | | 0.53 | |  |
| Rmet_4834 | *ompP* | Putative outer membrane pore protein (gram-negative type) | 8.02 | | 0.61 | |  |
| Rmet_2176 | Rmet_2176 | Conserved hypothetical protein | 5.51 | | 0.41 | |  |
| Rmet_4084 | *phoA1* | Alkaline phosphatase | 4.32 | | 0.89 | |  |
| Rmet_4085 | *phoA2* | Alkaline phosphatase | 3.37 | | 0.88 | |  |
| Rmet_0310 | Rmet_0310 | Putative intracellular protease/amidase/DJ-1/PfpI family | 2.76 | | 0.88 | |  |
| Rmet_0905 | Rmet_0905 | Putative metallo-dependent phosphoesterase | 2.73 | | 0.86 | |  |
| Rmet_0837 | Rmet_0837 | Hydroxamate-type ferrisiderophore receptor | 2.63 | | 0.95 | |  |
| Rmet_6085 | Rmet_6085 | Putative restriction endonuclease (fragment) | 2.58 | | NA | |  |
| Rmet_4726 | Rmet_4726 | Diguanylate cyclase/phosphodiesterase | 2.52 | | 1.25 | |  |
| Rmet_3407 | Rmet_3407 | Glycosyl transferase, family 2-protein | 2.51 | | 0.92 | |  |
| Rmet_1992 | *tnpA* | Transposase IS1087B | 2.51 | | 0.82 | |  |
| Rmet_0297 | Rmet_0297 | Probable nucleoside triphosphate hydrolase domain | 2.45 | | 1.06 | |  |
| Rmet_4727 | Rmet_4727 | Methyl-accepting chemotaxis sensory transducer | 2.36 | | 0.87 | |  |
| Rmet_0903 | *dgkA* | Diacylglycerol kinase | 2.35 | | 1.17 | |  |
| Rmet_3826 | Rmet_3826 | Conserved hypothetical protein | 2.27 | | 0.73 | |  |
| Rmet_1694 | Rmet_1694 | Conserved hypothetical protein | 2.17 | | 2.16 | |  |
| Rmet_6245 | Rmet_6245 | Conserved hypothetical protein (Orf95) | 2.17 | | 1.05 | |  |
| Rmet_2185 | *pstS* | Phosphate transporter subunit ; periplasmic-binding component of ABC superfamily | 2.15 | | 0.36 | |  |
| Rmet_6069 | Rmet_6069 | Sodium/hydrogen exchanger (Orf242) | 2.13 | | 1.11 | |  |
| Rmet_1200 | *phaP* | Phasin (PHA-granule associated protein) | 2.11 | | 0.92 | |  |
| Rmet_5910 | Rmet_5910 | Conserved hypothetical protein | 2.08 | | 0.76 | |  |
| Rmet_0572 | *cpdB* | 2':3'-cyclic-nucleotide 2'-phosphodiesterase | 2.06 | | 0.91 | |  |
| Rmet_0339 | Rmet_0339 | Monooxygenase, FAD-binding:FAD dependent oxidoreductase | 2.05 | | 0.96 | |  |
| Rmet_0388 | Rmet_0388 | Conserved hypothetical protein | 2.04 | | 1.03 | |  |
| Rmet_1512 | *cbbP* | Phosphoribulokinase | 2.02 | | 0.47 | |  |
| Rmet_1515 | *cbbG1* | Glyceraldehyde-3-phosphate dehydrogenase A (GAPDH-A) | 2.02 | | 0.45 | |  |
| Rmet_1514 | *cbbZ1* | Phosphoglycolate phosphatase (PGP) | 2.02 | | 0.40 | |  |
| Rmet_2070 | *argAB* | Acetylglutamate kinase and acetyltransferase | 2.00 | | 1.75 | |  |
| Rmet_0309 | Rmet_0309 | Conserved hypothetical protein | 2.00 | | 0.82 | |  |
| Rmet_0712 | *ompA* | Outer membrane protein or related peptidoglycan-associated (lipo)protein | 0.50 | | 0.92 | |  |
| Rmet_0171 | Rmet_0171 | Conserved hypothetical protein | 0.50 | | 0.83 | |  |
| Rmet_5232 | *cydA* | Cytochrome bd ubiquinol oxidase. subunit I | 0.50 | | 0.60 | |  |
| Rmet_0950 | *cyoC* | Cytochrome o ubiquinol oxidase subunit III | 0.49 | | 1.13 | |  |
| Rmet_0446 | *glyS* | Glycine tRNA synthetase. beta subunit | 0.49 | | 0.96 | |  |
| Rmet_5127 | Rmet_5127 | Putative sigma-54-dependent transcriptional regulator. HTH Fis-type family | 0.49 | | 0.96 | |  |
| Rmet_1420 | *dapE* | N-succinyl-diaminopimelate deacylase | 0.49 | | 1.21 | |  |
| Rmet_0348 | Rmet_0348 | ABC-type multidrug transporter. permease component | 0.49 | | 0.89 | |  |
| Rmet_0003 | *gyrB* | DNA gyrase. subunit B | 0.49 | | 0.93 | |  |
| Rmet_4603 | Rmet_4603 | Transcriptional regulator, GntR family | 0.49 | | 0.76 | |  |
| Rmet_1284 | *hypF1* | HypF1 hydrogenase maturation protein (CMGI-2) | 0.48 | | 0.67 | |  |
| Rmet_2149 | Rmet_2149 | Metallo-beta-lactamase family protein | 0.48 | | 0.78 | |  |
| Rmet_0469 | *sucC* | Succinyl-CoA synthetase, beta subunit | 0.48 | | 1.10 | |  |
| Rmet_4564 | *metE* | 5-methyltetrahydropteroyltriglutamate-homocysteine methyltransferase | 0.48 | | 0.53 | |  |
| Rmet_0799 | Rmet_0799 | Putative ABC-type transporter. permease component: QAT family | 0.48 | | 0.82 | |  |
| Rmet_1313 | *tmoD* | Toluene-4-monooxygenase system protein D | 0.48 | | 0.91 | |  |
| Rmet_0253 | Rmet_0253 | RNA-directed DNA polymerase (reverse transcriptase) | 0.48 | | 0.88 | |  |
| Rmet_2412 | *efp* | Translation elongation factor P (EF-P) | 0.47 | | 1.22 | |  |
| Rmet_1211 | Rmet_1211 | Putative ABC transporter, periplasmic substrate-binding protein | 0.47 | | 1.25 | |  |
| Rmet_0930 | *nuoD* | NADH-ubiquinone oxidoreductase D subunit (NADH dehydrogenase subunit D) | 0.47 | | 0.99 | |  |
| Rmet_2455 | *rpsU1* | 30S ribosomal subunit protein S21 | 0.47 | | 1.07 | |  |
| Rmet_1807 | Rmet_1807 | ADP-glucose pyrophosphorylase | 0.47 | | 1.13 | |  |
| Rmet_0401 | *gltK* | Glutamate and aspartate transporter subunit; membrane component of ABC superfamily | 0.47 | | 1.22 | |  |
| Rmet_0911 | *ilvI* | Acetolactate synthase III, large subunit | 0.47 | | 1.14 | |  |
| Rmet_2732 | *rfbC* | dTDP-4-deoxyrhamnose-3.5-epimerase | 0.47 | | 1.32 | |  |
| Rmet_2032 | *nusA* | Transcription termination/antitermination L factor | 0.46 | | 0.93 | |  |
| Rmet_4768 | *eda* | 2-keto-3-deoxy-phosphogluconate aldolase | 0.46 | | 0.91 | |  |
| Rmet_0802 | Rmet_0802 | Putative ABC-type transporter glycine betaine/L-proline transporter. ATPase subunit . ATPase component: QAT family | 0.46 | | 0.91 | |  |
| Rmet_1324 | *dmpB* | Metapyrocatechase (MPC) (CatO2ase) (Catechol 2.3- dioxygenase) (C23O) (gene belongs to CMGI-2) | 0.46 | | ND | |  |
| Rmet_2451 | *fdx8* | Ferredoxin | 0.46 | | 1.12 | |  |
| Rmet_0929 | *nuoC* | NADH dehydrogenase chain C | 0.46 | | 1.06 | |  |
| Rmet_2137 | Rmet_2137 | 50S ribosomal protein L31 type B | 0.46 | | 1.23 | |  |
| Rmet_1435 | *rpsB* | 30S ribosomal subunit protein S2 | 0.46 | | 1.05 | |  |
| Rmet_3423 | *soxY* | Sulfur oxidation protein (SoxY) | 0.46 | | 1.04 | |  |
| Rmet_0680 | *nadE* | Glutamine-dependent NAD(+) synthetase (NAD(+) synthase [glutamine-hydrolyzing]) | 0.46 | | 0.78 | |  |
| Rmet_2460 | *purF* | Amidophosphoribosyltransferase | 0.46 | | 1.09 | |  |
| Rmet_1288 | *hoxT* | HoxT putative hydrogenase expression/formation protein (CMGI-2) | 0.45 | | 0.57 | |  |
| Rmet_4699 | Rmet_4699 | Conserved hypothetical protein; putative transmembrane protein | 0.45 | | 0.83 | |  |
| Rmet_0141 | Rmet_0141 | Pyrimidine 5-nucleotidase | 0.45 | | 0.84 | |  |
| Rmet_0826 | Rmet_0826 | Putative transmembrane protein; putative transporter, APC superfamily | 0.45 | | 0.96 | |  |
| Rmet_0114 | *bioA* | 7.8-diaminopelargonic acid synthase, PLP-dependent | 0.45 | | 0.94 | |  |
| Rmet_3292 | *rpsD* | 30S ribosomal subunit protein S4 | 0.45 | | 1.02 | |  |
| Rmet_2547 | Rmet_2547 | Conserved hypothetical protein | 0.45 | | 1.12 | |  |
| Rmet_1445 | *lpxD* | UDP-3-O-(3-hydroxymyristoyl)-glucosamine N-acyltransferase | 0.45 | | 0.92 | |  |
| Rmet_2102 | *der* | GTP-binding protein | 0.45 | | 1.08 | |  |
| Rmet_2730 | Rmet_2730 | Hypothetical protein; putative membrane protein | 0.44 | | 1.20 | |  |
| Rmet_0737 | *etfB* | Electron transfer flavoprotein subunit beta (Beta-ETF) (Electron transfer flavoprotein small subunit) (ETFSS) | 0.44 | | 1.08 | |  |
| Rmet_3496 | *atpA* | F1 sector of membrane-bound ATP synthase. alpha subunit | 0.44 | | 0.92 | |  |
| Rmet_0117 | *bioB* | Biotin synthase | 0.44 | | 0.88 | |  |
| Rmet_2726 | Rmet_2726 | Sugar transferase involved in lipopolysaccharidesynthesis | 0.43 | | 0.99 | |  |
| Rmet_1444 | *hlpA* | Periplasmic chaperone | 0.43 | | 1.11 | |  |
| Rmet_2452 | *trxB2* | Thioredoxin reductase | 0.43 | | 1.11 | |  |
| Rmet_2979 | *cbbG2* | Glyceraldehyde-3-phosphate dehydrogenase A (CbbG) | 0.43 | | 0.94 | |  |
| Rmet_0935 | *nuoI* | NADH:ubiquinone oxidoreductase, chain I | 0.43 | | 1.24 | |  |
| Rmet_3498 | *atpF* | F0 sector of membrane-bound ATP synthase, subunit b | 0.43 | | 1.03 | |  |
| Rmet_1285 | *hypB1* | HypB1 GTP hydrolase involved in nickel liganding into hydrogenases (CMGI-2) | 0.43 | | 0.67 | |  |
| Rmet_0941 | Rmet_0941 | Conserved hypothetical protein; putative membrane protein | 0.43 | | 1.06 | |  |
| Rmet_1210 | Rmet_1210 | ABC transporter, inner membrane subunit | 0.42 | | 1.16 | |  |
| Rmet_0172 | *metF* | 5.10-methylenetetrahydrofolate reductase | 0.42 | | 0.65 | |  |
| Rmet_0521 | *bug* | Extra-cytoplasmic Solute Receptor protein | 0.42 | | 1.20 | |  |
| Rmet_1584 | Rmet_1584 | Conserved hypothetical protein | 0.42 | | 0.63 | |  |
| Rmet_3497 | *atpH* | F1 sector of membrane-bound ATP synthase, delta subunit | 0.42 | | 1.10 | |  |
| Rmet_1886 | *tig* | Peptidyl-prolyl cis/trans isomerase (trigger factor) | 0.41 | | 1.06 | |  |
| Rmet_0722 | *rpsA* | 30S ribosomal subunit protein S1 | 0.41 | | 1.00 | |  |
| Rmet_1422 | Rmet_1422 | Conserved hypothetical protein | 0.41 | | 0.99 | |  |
| Rmet_0923 | *pnp* | Polynucleotide phosphorylase/polyadenylase | 0.41 | | 0.74 | |  |
| Rmet_3153 | Rmet_3153 | ABC-type branched-chain amino acid transport system. periplasmic component | 0.41 | | 0.80 | |  |
| Rmet_0913 | *ilvC* | Ketol-acid reductoisomerase | 0.41 | | 1.44 | |  |
| Rmet_5072 | Rmet_5072 | Conserved hypothetical protein; partial overlap | 0.41 | | 0.85 | |  |
| Rmet_1421 | *arsC3* | Putative arsenate reductase or related protein (glutaredoxin family) | 0.41 | | 1.05 | |  |
| Rmet_2273 | *fumA* | Fumarate hydratase class I | 0.41 | | 1.16 | |  |
| Rmet_0749 | *rimM* | 16S rRNA processing protein | 0.41 | | 0.75 | |  |
| Rmet_1163 | *rplT* | 50S ribosomal subunit protein L20 | 0.40 | | 1.04 | |  |
| Rmet_2484 | *sdhA* | Succinate dehydrogenase, flavoprotein subunit | 0.40 | | 1.42 | |  |
| Rmet_0130 | *bug* | Extra-cytoplasmic Solute Receptor | 0.40 | | 1.01 | |  |
| Rmet_1443 | *yaeT* | Outer membrane protein assembly factor.outer membrane protein. surface antigen OMA87 | 0.40 | | 1.10 | |  |
| Rmet_3316 | *rplD* | 50S ribosomal subunit protein L4 | 0.40 | | 1.23 | |  |
| Rmet_2473 | *leuD* | 3-isopropylmalate isomerase subunit | 0.39 | | 1.34 | |  |
| Rmet_1583 | *ansB* | Periplasmic L-asparaginase II | 0.39 | | 1.46 | |  |
| Rmet_2727 | Rmet_2727 | Putative glycosyl transferase. group 1 | 0.38 | | 1.01 | |  |
| Rmet_1977 | *rpsR* | SSU ribosomal protein S18 | 0.38 | | 1.25 | |  |
| Rmet_0411 | *rpsI* | 30S ribosomal subunit protein S9 | 0.38 | | 1.04 | |  |
| Rmet_2697 | Rmet_2697 | Transglycosylase SLT domain protein | 0.38 | | 0.93 | |  |
| Rmet_4773 | Rmet_4773 | Putative endoribonuclease L-PSP | 0.38 | | 1.35 | |  |
| Rmet_0932 | *nuoF* | NADH:ubiquinone oxidoreductase. chain F | 0.38 | | 1.04 | |  |
| Rmet_1162 | *rpmI* | 50S ribosomal subunit protein L35 | 0.38 | | 0.88 | |  |
| Rmet_1298 | *hoxK* | HoxK membrane-bound [NiFe] hydrogenase small subunit (belongs to CMGI-2) | 0.38 | | 0.90 | |  |
| Rmet_4286 | *bug* | Extra-cytoplasmatic solute receptor | 0.38 | | 1.18 | |  |
| Rmet_0932 | *nuoF* | NADH:ubiquinone oxidoreductase, chain F | 0.38 | | 1.11 | |  |
| Rmet_3290 | *rplQ* | 50S ribosomal subunit protein L17 | 0.37 | | 1.02 | |  |
| Rmet_5074 | Rmet_5074 | Conserved hypothetical protein | 0.37 | | 0.99 | |  |
| Rmet_3494 | *atpD* | F1 sector of membrane-bound ATP synthase. beta subunit | 0.37 | | 0.81 | |  |
| Rmet_1290 | *hoxQ* | HoxQ protein involved in nickel incorporation into hydrogenase-1 proteins(belongs to CMGI-2) | 0.37 | | 0.68 | |  |
| Rmet_0115 | *bioF* | 8-amino-7-oxononanoate synthase | 0.37 | | 0.79 | |  |
| Rmet_1979 | *rpsF* | 30S ribosomal subunit protein S6 | 0.37 | | 1.17 | |  |
| Rmet_0089 | Rmet_0089 | Hypothetical protein | 0.37 | | 0.94 | |  |
| Rmet_0931 | *nuoE* | NADH dehydrogenase chain E | 0.36 | | 1.02 | |  |
| Rmet_0940 | *nuoN* | NADH:ubiquinone oxidoreductase, membrane subunit N | 0.35 | | 1.07 | |  |
| Rmet_3333 | *rpoC* | RNA polymerase, beta prime subunit | 0.35 | | 0.89 | |  |
| Rmet_3106 | *rplU* | 50S ribosomal subunit protein L21 | 0.35 | | 0.91 | |  |
| Rmet_2485 | *sdhD* | Succinate dehydrogenase, membrane subunit, binds cytochrome b556 | 0.34 | | 1.23 | |  |
| Rmet_2481 | *gltA* | Citrate synthase | 0.34 | | 1.21 | |  |
| Rmet_1011 | Rmet_1011 | Alpha/beta hydrolase superfamily | 0.34 | | 0.64 | |  |
| Rmet_1446 | *fabZ* | (3R)-hydroxymyristol acyl carrier protein dehydratase | 0.33 | | 0.94 | |  |
| Rmet_3323 | *rpsJ* | 30S ribosomal subunit protein S10 | 0.33 | | 1.06 | |  |
| Rmet_2814 | *cysH* | Phosphoadenosine phosphosulfate reductase | 0.33 | | 1.30 | |  |
| Rmet_2813 | *cysD* | Sulfate adenylyltransferase, subunit 2 | 0.33 | | 1.14 | |  |
| Rmet_3317 | *rplC* | 50S ribosomal subunit protein L3 | 0.32 | | 1.12 | |  |
| Rmet_3339 | *nusG* | Transcription termination factor | 0.32 | | 0.95 | |  |
| Rmet_1295 | *hoxZ* | HoxZ Probable membrane-bound Ni/Fe-hydrogenase-linked b-type cytochrome (belongs to CMGI-2) | 0.31 | | 0.57 | |  |
| Rmet_0934 | *nuoH* | NADH:ubiquinone oxidoreductase, membrane subunit H | 0.31 | | 1.00 | |  |
| Rmet_3495 | *atpG* | F1 sector of membrane-bound ATP synthase, gamma subunit | 0.31 | | 0.83 | |  |
| Rmet_0936 | *nuoJ* | NADH dehydrogenase chain J | 0.30 | | 1.11 | |  |
| Rmet_3334 | *rpoB* | RNA polymerase, beta subunit | 0.30 | | 0.95 | |  |
| Rmet_2486 | *sdhC* | Succinate dehydrogenase, membrane subunit. binds cytochrome b556 | 0.30 | | 1.18 | |  |
| Rmet_1161 | *infC* | Protein chain initiation factor IF-3 | 0.30 | | 0.76 | |  |
| Rmet_5816 | *cspA* | Major cold shock protein | 0.29 | | 0.66 | |  |
| Rmet_5075 | *msuE1* | NADPH-dependent FMN reductase | 0.29 | | 0.70 | |  |
| Rmet_2483 | *sdhB* | Succinate dehydrogenase, FeS subunit | 0.28 | | 1.25 | |  |
| Rmet_3303 | *rpsH* | 30S ribosomal subunit protein S8 | 0.28 | | 0.87 | |  |
| Rmet_2870 | *rpmB* | 50S ribosomal subunit protein L28 | 0.28 | | 1.03 | |  |
| Rmet_3338 | *rplK* | 50S ribosomal subunit protein L11 | 0.27 | | 0.95 | |  |
| Rmet_1976 | *rplI* | 50S ribosomal subunit protein L9 | 0.27 | | 1.29 | |  |
| Rmet_3293 | *rpsK* | 30S ribosomal subunit protein S11 | 0.27 | | 0.84 | |  |
| Rmet_0937 | *nuoK* | NADH:ubiquinone oxidoreductase, membrane subunit K | 0.27 | | 1.25 | |  |
| Rmet_3291 | *rpoA* | RNA polymerase, alpha subunit | 0.27 | | 1.15 | |  |
| Rmet_0116 | *bioD* | Dethiobiotin synthetase | 0.26 | | 0.86 | |  |
| Rmet_0410 | *rplM* | 50S ribosomal subunit protein L13 | 0.26 | | 1.00 | |  |
| Rmet_5073 | *metE* | 5-methyltetrahydropteroyltriglutamate-homocysteine methyltransferase | 0.26 | | 0.91 | |  |
| Rmet_3337 | *rplA* | 50S ribosomal subunit protein L1 | 0.26 | | 1.11 | |  |
| Rmet_3326 | *rpsG* | 30S ribosomal subunit protein S7 | 0.26 | | 1.03 | |  |
| Rmet_0288 | *rplY* | 50S ribosomal protein L25 (General stress protein CTC) | 0.25 | | 0.98 | |  |
| Rmet_3307 | *rplN* | 50S ribosomal subunit protein L14 | 0.25 | | 0.79 | |  |
| Rmet_0751 | *rplS* | 50S ribosomal subunit protein L19 | 0.25 | | 0.72 | |  |
| Rmet_3544 | Rmet_3544 | Putative chloramphenicol resistance permease | 0.24 | | 0.62 | |  |
| Rmet_3315 | *rplW* | 50S ribosomal subunit protein L23 | 0.24 | | 1.24 | |  |
| Rmet_3304 | *rpsN* | 30S ribosomal subunit protein S14 | 0.24 | | 1.00 | |  |
| Rmet_2482 | Rmet_2482 | Conserved hypothetical protein | 0.23 | | 1.21 | |  |
| Rmet_3306 | *rplX* | 50S ribosomal subunit protein L24 | 0.23 | | 0.80 | |  |
| Rmet_3300 | *rpsE* | 30S ribosomal subunit protein S5 | 0.22 | | 0.85 | |  |
| Rmet_2480 | *livK1* | Leucine/isoleucine/valine transporter subunit. periplasmic-binding component of ABC superfamily | 0.22 | | 1.01 | |  |
| Rmet_1294 | Rmet_1294 | Putative plasmid maintenance system antidote protein. XRE family Putative HTH-type transcriptional regulator ybaQ (belongs to CMGI-2) | 0.22 | | 0.69 | |  |
| Rmet_3298 | *rplO* | 50S ribosomal subunit protein L15 | 0.22 | | 0.86 | |  |
| Rmet_1978 | *priB* | Primosomal replication protein N (priB-like) | 0.21 | | 1.30 | |  |
| Rmet_3305 | *rplE* | 50S ribosomal subunit protein L5 | 0.21 | | 0.81 | |  |
| Rmet_3314 | *rplB* | 50S ribosomal subunit protein L2 | 0.21 | | 0.98 | |  |
| Rmet_3296 | *infA* | Translation initiation factor IF-1 | 0.21 | | 0.87 | |  |
| Rmet_0939 | *nuoM* | NADH:ubiquinone oxidoreductase, membrane subunit M | 0.20 | | 1.01 | |  |
| Rmet_3308 | *rpsQ* | 30S ribosomal subunit protein S17 | 0.20 | | 0.91 | |  |
| Rmet_3297 | *secY* | Preprotein translocase membrane subunit | 0.19 | | 0.65 | |  |
| Rmet_3301 | *rplR* | 50S ribosomal subunit protein L18 | 0.19 | | 0.93 | |  |
| Rmet_3302 | *rplF* | 50S ribosomal subunit protein L6 | 0.19 | | 0.91 | |  |
| Rmet_3325 | *fusA1* | Protein chain elongation factor EF-G, GTP-binding | 0.19 | | 0.74 | |  |
| Rmet_3312 | *rplV* | 50S ribosomal subunit protein L22 | 0.18 | | 0.96 | |  |
| Rmet_3327 | *rpsL* | 30S ribosomal subunit protein S12 | 0.18 | | 0.81 | |  |
| Rmet_3324 | *tuf* | Protein chain elongation factor EF-Tu | 0.18 | | 0.94 | |  |
| Rmet_3299 | *rpmD* | 50S ribosomal subunit protein L30 | 0.17 | | 0.78 | |  |
| Rmet_3310 | *rplP* | 50S ribosomal subunit protein L16 | 0.17 | | 0.78 | |  |
| Rmet_3294 | *rpsM* | 30S ribosomal subunit protein S13 | 0.17 | | 0.80 | |  |
| Rmet_3313 | *rpsS* | 30S ribosomal subunit protein S19 | 0.16 | | 1.08 | |  |
| Rmet_3311 | *rpsC* | 30S ribosomal subunit protein S3 | 0.15 | | 0.99 | |  |
| Rmet_3309 | *rpmC* | 50S ribosomal subunit protein L29 | 0.14 | | 0.83 | |  |
| Rmet_3335 | *rplL* | 50S ribosomal subunit protein L7/L12 | 0.09 | | 0.76 | |  |
| **C)** |  |  |  |  | |  |  |
| **Rmet** | **Gene** | **Protein Function** | **R^2^** | **R^3^** | |  |  |
| Rmet_0616 | *groL* | Cpn60 chaperonin GroEL. large subunit of GroESL | 0.77 | 4.34 | |  |  |
| Rmet_0150 | *pyrE* | Orotate phosphoribosyltransferase | 0.82 | 2.61 | |  |  |
| Rmet_4962 | Rmet_4962 | Putative diguanylate phosphodiesterase (EAL domain) | 0.96 | 2.61 | |  |  |
| Rmet_0615 | *groS* | Cpn10 chaperonin GroES, small subunit of GroESL | 0.65 | 2.50 | |  |  |
| Rmet_5947 | *pbrA* | P-type ATPase involved in Pb(II) resistance PbrA | 1.69 | 2.44 | |  |  |
| Rmet_0676 | Rmet_0676 | Conserved hypothetical protein | 1.84 | 2.30 | |  |  |
| Rmet_0970 | *ggt* | Gamma-glutamyltransferase 1 | 1.22 | 2.24 | |  |  |
| Rmet_6102 | *int* | Tyrosine-based site-specific recombinase(fragment) | 1.43 | 2.19 | |  |  |
| Rmet_6300 | *trbG* | Mating pair formation | 1.76 | 2.11 | |  |  |
| Rmet_2047 | Rmet_2047 | AFG1-like ATPase | 1.77 | 2.10 | |  |  |
| Rmet_4611 | Rmet_4611 | Conserved hypothetical protein | 1.30 | 2.09 | |  |  |
| Rmet_5634 | Rmet_5634 | Conserved hypothetical protein | 1.62 | 2.09 | |  |  |
| Rmet_6193 | *bimA1* | Tyrosine-based site-specific recombinase BimA | 1.51 | 2.06 | |  |  |
| Rmet_1991 | Rmet_1991 | Conserved hypothetical protein | 1.38 | 2.05 | |  |  |
| Rmet_3729 | *icdA* | Isocitrate dehydrogenase, NADP-dependent | 0.63 | 2.02 | |  |  |
| Rmet_2039 | Rmet_2039 | Conserved hypothetical protein | 1.30 | 0.49 | |  |  |
| Rmet_2085 | Rmet_2085 | Conserved hypothetical protein | 1.00 | 0.49 | |  |  |
| Rmet_3587 | *acrR* | DNA-binding transcriptional repressor | 0.62 | 0.48 | |  |  |
| Rmet_3714 | Rmet_3714 | Conserved hypothetical protein | 0.92 | 0.48 | |  |  |
| Rmet_2083 | Rmet_2083 | 2-nitropropane dioxygenase, NPD | 1.06 | 0.48 | |  |  |
| Rmet_1676 | *uspA4* | Universal stress protein, UspA family | 1.14 | 0.48 | |  |  |
| Rmet_1512 | *cbbP* | Phosphoribulokinase | 2.02 | 0.47 | |  |  |
| Rmet_5670 | *copB2* | Copper resistance protein B | 0.94 | 0.46 | |  |  |
| Rmet_4395 | *uspA9* | Universal stress protein UspA | 0.98 | 0.46 | |  |  |
| Rmet_1515 | *cbbG1* | Glyceraldehyde-3-phosphate dehydrogenase A (GAPDH-A) | 2.02 | 0.45 | |  |  |
| Rmet_1387 | *uspA3* | Universal stress protein, UspA family | 1.20 | 0.45 | |  |  |
| Rmet_1677 | *uspA5* | Universal stress protein, UspA family | 0.97 | 0.44 | |  |  |
| Rmet_2180 | *phoB* | DNA-binding response regulator in two-component regulatory system with PhoR (or CreC) | 1.70 | 0.41 | |  |  |
| Rmet_2182 | *pstB* | Phosphate transporter subunit; ATP-binding component of ABC superfamily | 1.57 | 0.41 | |  |  |
| Rmet_4858 | Rmet_4858 | Conserved hypothetical protein | 1.17 | 0.41 | |  |  |
| Rmet_2211 | *ctpF* | Cation-transporting ATPase F | 1.21 | 0.40 | |  |  |
| Rmet_1514 | *cbbZ1* | Phosphoglycolate phosphatase (PGP) | 2.02 | 0.40 | |  |  |
| Rmet_3170 | *NirJ* | Heme d1 biosynthesis protein NirJ | 0.96 | 0.40 | |  |  |
| Rmet_0689 | Rmet_0689 | Conserved hypothetical protein | 0.87 | 0.39 | |  |  |
| Rmet_2183 | *pstA* | Phosphate transporter subunit; membrane component of ABC superfamily | 1.31 | 0.39 | |  |  |
| Rmet_4452 | Rmet_4452 | Response regulator receiver domain protein (CheY-like) | 1.22 | 0.39 | |  |  |
| Rmet_3474 | *hemN* | Coproporphyrinogen III oxidase. SAM and NAD(P)H dependent, oxygen-independent | 0.84 | 0.37 | |  |  |
| Rmet_0594 | *iorA* | Isoquinoline 1-oxidoreductase subunit alpha | 0.80 | 0.37 | |  |  |
| Rmet_2185 | *pstS* | Phosphate transporter subunit; periplasmic-binding component of ABC superfamily | 2.15 | 0.36 | |  |  |
| Rmet_0888 | Rmet_0888 | Fatty acid desaturase | 1.13 | 0.36 | |  |  |
| Rmet_2181 | *phoU* | Negative regulator of PhoR/PhoB two-component regulator | 1.93 | 0.34 | |  |  |
| Rmet_5671 | *copA2* | Copper resistance protein A, multi-copper oxidase | 0.99 | 0.34 | |  |  |
| Rmet_2155 | Rmet_2155 | Cytochrome c family protein | 0.92 | 0.33 | |  |  |
| Rmet_4761 | Rmet_4761 | Conserved hypothetical protein | 1.39 | 0.32 | |  |  |
| Rmet_2040 | *rdxB* | 4Fe-4S ferredoxin. iron-sulfur binding | 0.90 | 0.32 | |  |  |
| Rmet_5978 | *czcR* | Regulator, two components regulatory system involved in Cd(II), Zn(II) and Co(II) resistance | 0.90 | 0.32 | |  |  |
| Rmet_0458 | *uspA1* | Universal stress protein, UspA family | 0.86 | 0.31 | |  |  |
| Rmet_5645 | *adh* | Alcohol dehydrogenase, zinc-binding | 1.30 | 0.30 | |  |  |
| Rmet_2046 | *ccoI* | Copper-translocating P-type ATPase | 0.58 | 0.28 | |  |  |
| Rmet_2087 | *narL* | Transcriptional regulator, *luxR* family | 0.68 | 0.25 | |  |  |
| Rmet_3522 | *pldB* | Lysophospholipase | 1.18 | 0.23 | |  |  |
| Rmet_3523 | *cupR* | CupR transcriptional activator (MerR family) | 0.77 | 0.21 | |  |  |
| Rmet_3524 | *cupA* | P-type ATPase CupA | 1.14 | 0.21 | |  |  |
| Rmet_4521 | Rmet_4521 | Transcriptional regulator, Crp/Fnr family | 1.08 | 0.20 | |  |  |
| Rmet_2041 | *ccoP* | Cbb3-type cytochrome oxidase. diheme subunit IV | 0.84 | 0.19 | |  |  |
| Rmet_3172 | *nirS* | Nitrite reductase precursor (Cytochrome cd1) (Cytochrome oxidase) | 0.92 | 0.19 | |  |  |
| Rmet_2043 | *ccoO* | Cbb3-type cytochrome oxidase, monoheme subunit II | 0.93 | 0.18 | |  |  |
| Rmet_5886 | Rmet_5886 | Outer membrane protein, OmpW family | 0.77 | 0.18 | |  |  |
| Rmet_2042 | *ccoQ* | Cbb3-type cytochrome oxidase, subunit III | 0.71 | 0.17 | |  |  |
| Rmet_2044 | *ccoN* | Cbb3-type cytochrome oxidase, subunit I | 0.71 | 0.14 | |  |  |
| Rmet_3525 | *cupC* | Copper chaperone, heavy metal ion binding (modular protein) | 1.12 | 0.11 | |  |  |
