# Supplementary material for: Zinc-Induced Transposition of Insertion Sequence Elements Contributes to Increased Adaptability of Cupriavidus metallidurans
Source: Front Microbiol. 2016 Mar 23;7:359. doi: 10.3389/fmicb.2016.00359 (PMC4803752; doi:10.3389/fmicb.2016.00359)
Supplement: Supplementary Table 3 — Scan of the C. metallidurans CH34 genome sequence (+ for forward and – for reverse strand) with the position-specific scoring matrix obtained from the multiple sequence alignment of the ISRme5 target sites in 87 zinc-resistant AE126 derivatives using RSAT. The weight score Ws = log (P(S|M)/P(S|B)) is the probability for the sequence segment S to occur according to the motif model M, whereas P (S|B) is the probability for the same sequence S to occur under the background model B. The weight score is thus the log-ratio of the likelihood of S in these two respective models. Top 100 targets are shown. [file Table3.DOCX]

| Supplementary Table 3. Scan of the *C*. *metallidurans* CH34 genome (+ for forward and – for reverse strand) with the profile matrix (Figure 5) obtained from the six different insertion sites of IS*Rme5* in *cnrYX* identified in 87 zinc-resistant AE126 derivatives using RSAT. The weight score (W_s_= log (P(S\|M)/P(S\|B)) is the probability for the sequence segment S to occur according to the motif model M, whereas P (S\|B) is the probability for the same sequence S to occur under the background model B. The weight score is thus the log-ratio of the likelihood of S in these two respective models. Top 100 targets are shown. | | | | |
| --- | --- | --- | --- | --- |
| **Rmet** | **Protein Function** | **Strand** | **Sequence** | **Score** |
| Rmet_6206 | CnrX, antisigma factor, regulatory protein, involved in Co(II) and Ni (II) response | + | GCTGTTCCACTAGATGCCAACGAGCG | 27.4 |
| Rmet_6206 | CnrX, antisigma factor, regulatory protein, involved in Co(II) and Ni (II) response | + | GCTTTCCTTGTCTACGCTGTTTGGCG | 18.9 |
| Rmet_0375 | Putative Na+/solute symporter | - | GCTGGTGTTCTATACGGTGTCGGACG | 14.2 |
| Rmet_3403 | Proline/glycine betaine transporter: Permease of the major facilitator superfamily (MFS) | + | GCTGGTGTTCTACATGCCGTCCTACG | 13.5 |
| Rmet_0591 | Putative signal transduction protein containing a membrane domain, an EAL and a GGDEF domain | + | GATGTCCCAGTTCGTGCCGACCAGCG | 12.9 |
| Rmet_3395 | Conserved hypothetical protein | + | GATGTAGTTGCCGATGCCACCGAGCG | 12.3 |
| Rmet_1019 | Excinuclease of nucleotide excision repair, DNA damage recognition component | + | CCTGTCGTACCAGACGCTGCTGGGCG | 12 |
| Rmet_0858 | Bifunctional (p)ppGpp synthetase II and guanosine-3',5'-bis pyrophosphate 3'-pyrophosphohydrolase | + | CATCTTGCGGTAGATGCTGTAGAGCG | 11.7 |
| Rmet_4666 | Hydrogenase-4 subunit I | + | GATGTTGTAGTAGGGGTTGCCGAGCG | 11.2 |
| Rmet_5141 | Acyl-CoA dehydrogenase | + | GCTGCCGTACTTCATGATGACGGGCG | 10.7 |
| Rmet_2522 | Major facilitator superfamily MFS_1 precursor | - | GAAGTACCTGTCGATGCTGTCGGGTG | 10.7 |
| Rmet_3661 | Conserved hypothetical protein | - | GTTGTTGCAGTCGACGCCGTTGAGCC | 10.6 |
| Rmet_0880 | Putative outer membrane protein (porin) | + | GCTGCTGTACTCGACGCCGAACTTCG | 10.6 |
| Rmet_1479 | Conserved hypothetical protein | - | GCTGGTCTACTGGACGATGACTTATG | 10.1 |
| Rmet_2946 | SecD, SECYEG protein translocase auxiliary subunit | - | GGTGCTGTACTACATGCTGTTCGGCG | 10.1 |
| Rmet_3779 | Putative monooxygenase, FAD/NAD(P)-binding domain | + | GCTGTTCCTGTCGATCCTGCTCAAGG | 10 |
| Rmet_2979 | Glyceraldehyde-3-phosphate dehydrogenase A (CbbG) | + | GATCTTCTTGCCGTTGACCACGAGCG | 9.9 |
| Rmet_3233 | Trypsin-like serine protease | + | GCTTGCCTACTCTTCCTCATCCAGCG | 9.7 |
| Rmet_1222 | Enoyl-CoA hydratase/isomerase | - | GTCTTCCCAGTCGATGTCGTCGAGCG | 9.7 |
| Rmet_5315 | Putative major facilitator superfamily transporter | + | GCTGGTGCTGCTGTTGCTGTTGGGCG | 9.7 |
| Rmet_4468 | Heavy metal cation tricomponent efflux pump CzcA2 | - | GATGTTCCACCCGATGGCGATGACCG | 9.5 |
| Rmet_1819 | TonB-dependent siderophore receptor | + | GCGGTCCTGGTAGATGCCGTCGAAGG | 9.5 |
| Rmet_5752 | Conserved hypothetical protein | - | GCTGGCCTACGAGAGGATGATGGGCG | 9.3 |
| Rmet_5849 | Tyrosine-protein kinase | - | GCTTTACCAGTCCTTGCTCAATGACG | 9.2 |
| Rmet_3561 | Conserved hypothetical protein | - | GCAGTTCTCGCAAACGCTGTCTAACG | 9.2 |
| Rmet_1970 | PhoH family protein | - | GCTGTTCTTCCAGACGCGCCTGAACG | 9.2 |
| Rmet_0518 | Conserved hypothetical protein | + | CATTTTCTTCTTCGTGCCGAAGGGCG | 9 |
| Rmet_4529/4530 | Inter: Sulfatase-modifying factor/conserved hypothetical protein | + | GATTTTTTACAAGATTCTTAATAGCG | 9 |
| Rmet_3966 | Hypothetical protein | - | GCTCCTGCTGTTTACGCCAACCAACC | 8.8 |
| Rmet_5890 | Fe^2+^ transport system protein B | - | CCTGTTCCTGTTTATCCTGACGCTCG | 8.8 |
| Rmet_4192 | Phospholipase C signal peptide protein | + | GCTGTTCGGGTTGATGAAGTCGAACG | 8.8 |
| Rmet_0600 | Multidrug efflux system, subunit B | + | GCAGTACCAGTACACGCTCACCGACG | 8.7 |
| Rmet_0667 | HemL, glutamate-1-semialdehyde aminotransferase | - | GCCTGCCTGATAGACGCCACCGAGCG | 8.6 |
| Rmet_1619 | Conserved hypothetical protein | + | GCTGCTGCTGCAGACGCCGGAGGGCG | 8.6 |
| Rmet_4165 | Lipoprotein VacJ precursor | - | GCTATCCCGCTTTTCGCCTTCGGGCG | 8.5 |
| Rmet_5158/5159 | Inter: Acyl-CoA ligase/Transcriptional regulator, LysR family | - | GCTGGCGTTCTTGATGACGTTTTGCC | 8.5 |
| Rmet_4700 | Conserved hypothetical protein | - | GCTTATCGAGCCGACGCTGATGGGCG | 8.4 |
| Rmet_3404 | Tag, 3-methyl-adenine DNA glycosylase I, constitutive | + | GGCTTCCCTGTCGACGACGACCGGCG | 8.4 |
| Rmet_2447 | Na+/H+ antiporter NhaD or related arsenite permease transmembrane protein | + | GCTGTCCGAGTACATCCCGTTCATCG | 8.4 |
| Rmet_2185 | PstS, phosphate transporter subunit; periplasmic-binding component of ABC syperfamily | - | GTCGTTCTACCAGATCCTGACGAACG | 8.3 |
| Rmet_2999 | Propeptide, PepSY and peptidase M4 precursor (Tn*6048*) | - | GATGTCCGGGTCGATGCCGACAAGGG | 8.1 |
| Rmet_2525 | AMP-dependent synthetase and ligase | + | GCTGGACCTGTCGTCGCTGAACAGCC | 8.1 |
| Rmet_2999 | Propeptide, PepSY and peptidase M4 precursor (Tn*6048*) | - | GATGTCCGGGTCGATGCCGACAAGGG | 8.1 |
| Rmet_5488 | Conserved hypothetical protein | + | GCTCGTCCTCTAGAGGCTCACCTAGG | 8.1 |
| Rmet_2999 | Propeptide, PepSY and peptidase M4 precursor (Tn*6048*) | + | GATGTCCGGGTCGATGCCGACAAGGG | 8.1 |
| Rmet_5614 | Putative CDP-diacylglycerol pyrophosphatase | + | GACGTTCTTCCAGAGGCTGTCGCGCG | 8 |
| Rmet_0876 | Aminopeptidase N | - | GCCGTCCTGCTTGACGCCGGAGAACG | 7.9 |
| Rmet_2176 | Hemolysin-like Acyl-CoA N-acyltransferase | + | GCTCTTCGACTCCTTGCCCACTGGCC | 7.9 |
| Rmet_2069 | ATP-dependent helicase | + | GCTGGCCTTCCAGATCCTGATAGGCC | 7.8 |
| Rmet_0797 | Sugar ATP binding ABC transporter protein; ATPase component: CUT2 family | + | GCTCTCTCACCAGACGCCGCCGTTCG | 7.8 |
| Rmet_1389 | Predicted hydrolase (HAD superfamily) | - | GCTGTTCGACCACGTGCTGTCGGTCG | 7.8 |
| Rmet_0170 | Adenosylhomocysteinase | + | GCCTTCCTTCTTCACGCCGATGTAGG | 7.8 |
| Rmet_1937 | Amidase | - | GCTGACCCACTGCATGCCACTGTGCG | 7.8 |
| Rmet_4163 | Conserved hypothetical protein, peptidoglycan-binding LysM domain, putative membrane protein | - | GCCGCCCTTGTCGATGCCGATCATCG | 7.8 |
| Rmet_5805 | Putative major facilitator superfamily (MFS) transporter | - | GCTGGTGCAGTTTCTGCCGTCGATCG | 7.8 |
| Rmet_4823 | Conserved hypothetical protein | - | GCTGGCGTCCTTGATGCCGATCGACG | 7.7 |
| Rmet_0659/0660 | Inter: Response regulator containing CheY-like receiver, AAA-type ATPase, and DNA-binding domains; Sigma-54 dependent DNA-binding transcriptional regulator/Transcriptional regulator, LysR family | + | GCTGTTGCGCCAGATGCCACCACGCG | 7.7 |
| Rmet_2671 | Putative YeeE-like protein | + | GCTCTCGGCCTATATGACGTTGCGCG | 7.7 |
| Rmet_2591 | Transcriptional regulator, DeoR family | - | GAATGCCTTGTCGACGCTGATGCGCG | 7.6 |
| Rmet_2776 | GCN5-related N-acetyltransferase | - | GCTGTCCGACTCTACGCCTCCCAGGC | 7.6 |
| Rmet_0375 | Putative Na^+^ solute symporter | - | GATGATCCAATAGATGATGACGAACC | 7.6 |
| Rmet_3536 | Sensor histidine kinase | - | GATGTTGTTCGCCACGCCATACAGCG | 7.6 |
| Rmet_1252 | Protein involved in ICE excision | - | GCTGTCCTGCTCAGGGCCGTTTTGCG | 7.5 |
| Rmet_0517 | Putative membrane protein | - | GCCGTTCTTCTATGTACTGATGAATG | 7.5 |
| Rmet_0804 | Putative membrane protease subunit, stomatin/prohibitin-like transmembrane protein | - | CATCGTCTTCCCGTTGCCAATGGGCG | 7.5 |
| Rmet_4328 | Alpha/beta hydrolase fold | + | GCTTTCGCCGAGTACGCCAACGAGCG | 7.5 |
| Rmet_4073 | Bug, extra-cytoplasmic solute receptor protein | - | CCTTGACCACTTTGTGCCAACGGTCG | 7.5 |
| Rmet_5233 | CydB, cytochrome d ubiquinol oxidase, subunit II | + | GATGGGCTTGTCGATGCTGATCTACG | 7.4 |
| Rmet_2750 | Phosphoenolpyruvate carboxylase | - | GCTGCCGCTGTCGTCGCTGATGGTCG | 7.4 |
| Rmet_0942 | Putative ADP-ribose pyrophosphatase | + | GATTTCCTACTCGACCGAGTTTATCG | 7.4 |
| Rmet_0600 | Multidrug efflux system, subunit B | + | GCTGTCGCAGATGACGTCGACCAGCG | 7.4 |
| Rmet_3218 | Putative Na^+^ driven multidrug efflux pump | - | GCTGTTCTACCGGATTCTCTCCATCG | 7.4 |
| Rmet_5826 | Acetyl-CoA C-acyltransferase | + | GCAGTTGTTGTTGACGTTGATGACCG | 7.3 |
| Rmet_4547 | Outer membrane porin signal peptide protein | + | GCTCGTCGTCACAACGCCATAGAGCG | 7.3 |
| Rmet_5084 | Member of cupin superfamily (RmlC-like) | - | GATTTTCTCGCCCACGACGTTGAGCG | 7.2 |
| Rmet_5191 | Amino acid permease | - | GGTGTTCTTCTACCTGCTGACGCTCG | 7.2 |
| Rmet_4867/4868 | Inter: Multidrug transport protein/esterase | - | GCTTTTCCAGTTGAAGAAACCCGACG | 7.2 |
| Rmet_0654 | Conserved hypothetical protein | + | GCTGTTCCTGTTCACGGTGTTGATGG | 7.2 |
| Rmet_1377 | Sulfate/thiosulfate transporter subunit; membrane component of ABC superfamily | + | GCCCTTCCAGTTGTTCCCGGCTGGCG | 7.2 |
| Rmet_0500 | Glutamyl-tRNA(Gln) amidotransferase subunit C | - | GCTCTCGCACTCTCCGACGTCAAGCG | 7.2 |
| Rmet_1713 | Putative membrane protein, putative ion channel | - | CATGTTGCTCTTGATGACATCGAGCA | 7.2 |
| Rmet_4605 | Histidine protein kinase | - | GCGCTTCCAGTCGACGCTGATGGATG | 7.2 |
| Rmet_4512 | Outer membrane efflux protein | + | GATTTTCCCGCTGATGCTGCTCGGCG | 7.1 |
| Rmet_4700 | Conserved hypothetical protein | + | GATGTCCCAGTACACCGCTTTGGGCG | 7.1 |
| Rmet_2808 | Predicted permease | - | GCTGTTCATCTCGACGCTGATCGTCC | 7.1 |
| Rmet_0326 | Hypothetical protein | + | GCCGATACTCTCTATGTCAAATGGCG | 7.1 |
| Rmet_0528 | Putative DNA-binding transcriptional regulator (LysR-family) | - | GCCGTCCTTCTCGTTGCCGTTGCCCG | 7 |
| Rmet_1705 | Conserved hypothetical protein | + | GCTGCCCTTCTTGAGGCTAACGGTCA | 7 |
| Rmet_1165 | Phenylalanine tRNA synthetase, beta subunit | - | GCTTTTCTCGTCAGCGCCAATGGCCG | 7 |
| Rmet_1647 | Conserved hypothetical protein | - | GCCGTTCCCGTAGAAGCCGTCTGGTG | 7 |
| Rmet_2973 | Leucyl-tRNA synthetase | - | GCTGTTCCTGAAGATGCTGGAAAAGG | 7 |
| Rmet_3991 | Arsenical pump membrane protein, putative arsenite permease | + | GATGTTGCTCGTGATGCCGACGAGCA | 7 |
| Rmet_5563 | Conserved hypothetical protein | + | GCCGTCCCGCTGGACGATGCCGAGCG | 7 |
| Rmet_5459 | Transposase Tn*7*-like proteinA | + | GCTTTTCTTCCGTACGTTGTTCTTCG | 7 |
| Rmet_1671 | Poly(3-hydroxybutyrate) polymerase | + | GATGTAGTACTTGTTGATGCAAGGCG | 6.9 |
| Rmet_1718 | AMP-dependent synthetase and ligase | + | GCAGATCCTGTACACGCTGAATGACG | 6.8 |
| Rmet_0271 | Cytochrome oxidase assembly protein, Sco1/SenC family | + | GATGGCCCAGTATGTGCCGGCCTTCG | 6.8 |
| Rmet_3428 | Conserved hypothetical protein | + | GCCGGTCCAGTCGGTGCCGGGTAGCG | 6.8 |
| Rmet_0335 | Putative transglycosylase | - | GCTTTCGTAGTAGCTGCTGCTGGGGC | 6.8 |
| Rmet_2866 | Leucine/isoleucine/valine transporter subunit, membrane component of ABC superfamily | + | GCAGTTCTGGCCTATACCATCGAACG | 6.8 |
