# Supplementary material for: Zinc-Induced Transposition of Insertion Sequence Elements Contributes to Increased Adaptability of Cupriavidus metallidurans
Source: Front Microbiol. 2016 Mar 23;7:359. doi: 10.3389/fmicb.2016.00359 (PMC4803752; doi:10.3389/fmicb.2016.00359)
Supplement: Supplementary Table 4 — Transcriptional expression of the cnrCBAT cluster, determined by whole-microarray analysis, in two zinc-resistant AE126 derivatives (AE126R2 and AE126R3) and in a zinc-resistant AE126 ΔcnrH::tet derivative (AE126ΔcnrH::tetR1). Data are represented as fold change relative to the parental AE126 and AE126 ΔcnrH::tet, respectively. The full description of the microarray data have been deposited at the Gene Expression Omnibus website (http://www.ncbi.nlm.nih.gov/geo/) under accession number GSE74091. [file Table4.DOCX]

| Supplementary Table 4. Transcriptional expression of the *cnrCBAT* cluster, as determined by whole-microarray analysis, in two zinc-resistant AE126 derivatives (AE126^R2^ and AE126^R3^) and in a zinc-resistant AE126 *∆cnrH::tet* derivative (AE126∆*cnrH::tet*^R1^). Data are represented as fold change relative to the parental AE126 and AE126 *∆cnrH::tet*, respectively. The full description of the microarray data have been deposited at the Gene Expression Omnibus website (http://www.ncbi.nlm.nih.gov/geo/) under accession number GSE74091. | | | | | |
| --- | --- | --- | --- | --- | --- |
| **Rmet** | **Gene** | **Protein Function** | **R^2^** | **R^3^** | **∆R^1^** |
| Rmet_6208 | *cnrC* | Outer membrane protein, three components cation proton antiporter efflux system. involved in Co(II) and Ni(II) resistance | 6.29 | 10.19 | 0.60 |
| Rmet_6209 | *cnrB* | Membrane fusion protein. three components cation proton antiporter efflux system. involved in Co(II) and Ni(II) resistance | 11.77 | 20.48 | 2.12 |
| Rmet_6210 | *cnrA* | Inner membrane efflux pump, three components cation proton antiporter efflux system. involved in Co(II) and Ni(II) resistance | 9.68 | 21.69 | 2.05 |
| Rmet_6211 | *cnrT* | Cation Diffusion Facilitator, involved in Co(II) and Ni(II) resistance | 8.06 | 18.78 | 2.41 |
